# Exclusion of RNA-binding domains from G-quadruplex condensates by G-quadruplex ligands

**DOI:** 10.1039/d6cb00020g

**Published:** 2026-07-01

**Authors:** Yoshiki Hashimoto, Ryosuke Suzuki, Mizuho Aya, Nagisa Takamiya, Mitsuki Tsuruta, Takeru Torii, Toshiyuki Goto, Keiko Kawauchi, Daisuke Miyoshi

**Affiliations:** a Frontiers of Innovative Research in Science and Technology, Konan University 7-1-20 Minatojima-minamimachi Chuo-ku Kobe Hyogo 650-0047 Japan miyoshi@konan-u.ac.jp; b School of Physical and Mathematical Sciences, Nanyang Technological University Singapore 637371 Singapore; c Graduate School of Science, Technology and Innovation, Kobe University Rokko Nada-ku Kobe 657-8501 Japan

## Abstract

Biomolecular liquid–liquid phase separation, which forms droplets in living cells, plays a crucial role in the regulation of gene expression. Dysfunction of liquid–liquid phase separation leads to aberrant aggregates that sequester RNA-binding proteins, thereby impairing their functions, which potentially leads to the onset of neurodegenerative diseases. Preventing and reversing the sequestration of RNA-binding proteins from droplets represent a promising therapeutic approach. Noteworthily, the G-quadruplex, a non-canonical secondary structure of nucleic acids formed by a guanine-rich sequence, is an essential structural motif that triggers liquid–liquid phase separation with a partner protein. Thus, a G-quadruplex ligand, which selectively binds and stabilizes the G-quadruplex, can be promising for controlling liquid–liquid phase separation of G-quadruplexes and partner proteins. In this study, we investigated the effects of G-quadruplex ligands on the liquid–liquid phase separation of RNA G-quadruplexes and RGG domain-derived cationic peptides. It was found that G-quadruplex ligands formed aggregates with the target G-quadruplexes and excluded the G-quadruplex-binding peptides from these aggregates. Moreover, structure-selective G-quadruplex ligands induced aggregates only with the G-quadruplex but not with other secondary structures. These findings demonstrate for the first time that the structure-selectivity of G-quadruplex ligands plays a key role in modulating condensates.

## Abstract 2

Schematic illustration of peptide exclusion from G-quadruplex droplets induced by G4 ligands.

## Graphical abstract

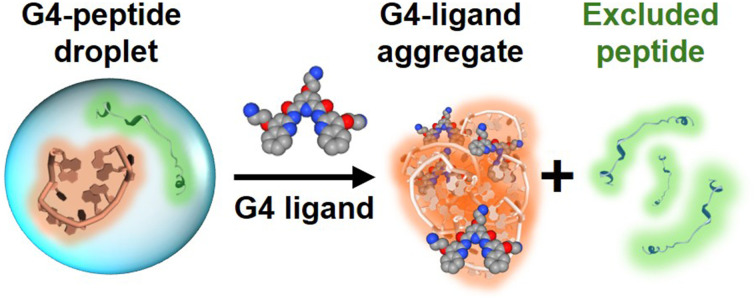

## Introduction

Biomolecular liquid–liquid phase separation (LLPS) mediates the formation of membraneless organelles and plays a central role in the spatiotemporal regulation of biomolecules within cells. One of the biological processes regulated by LLPS is gene expression. LLPS-dependent assemblies such as processing bodies and stress granules are known to regulate translation and transcription.^1–3^ In addition, LLPS enables cooperative and robust transcriptional bursting.^4–6^ Most LLPS involved in gene expression includes nucleic acids as components.^7^ Disruption of LLPS results in the formation of aberrant condensates that sequester RNA-binding proteins (RBPs), leading to the onset of neurodegenerative diseases.^8–11^ For example, abnormal expansions of GGC repeats in the *FMR1* gene pathological RBP are implicated in the fragile X syndrome (FXS) and fragile X-associated tremor/ataxia syndrome (FXTAS).^12^ Similarly, GGGGCC repeats in the *C9orf72* gene pathological RBP have been implicated in amyotrophic lateral sclerosis (ALS) and frontotemporal dementia (FTD).^12,13^ RBPs such as hnRNP, CUGBP1, and TRA2A are indispensable for splicing and translational regulation. If these proteins are sealed into aberrant condensates, they fail to maintain their functions, thereby causing defects in synaptic gene expression and neurotransmitter receptor regulation.^14–18^ Small molecules have been explored as inhibitors of aberrant aggregation. A europium complex has been shown to co-assemble with low molecular weight amyloid β (Aβ) oligomers and form non-fibrillar, degradable, non-toxic co-aggregates.^19^ (−)-Epigallocatechin gallate was found to inhibit the fibrillogenesis of both α-synuclein and Aβ efficiently, while concomitantly promoting the generation of a novel class of non-toxic α-synuclein and Aβ oligomers.^20^ Most proteins involved in LLPS are enriched in intrinsically disordered regions (IDRs),^21^ which adopt amorphous and dynamic structures, thus limiting the feasibility of selective inhibition by small molecules. This limitation highlights the urgent need for structured targets to replace IDRs.^22,23^

One of the most promising structured targets to control LLPS is the G-quadruplex (G4), a non-canonical secondary structure formed by guanine-rich nucleic acid sequences. For example, the GGC repeat sequence within the *FMR1* gene is known to fold into a G4.^24^ G4s have been suggested to participate in LLPS through interactions with RBPs. A wide variety of G4-targeting small molecules (G4 ligands) have been developed to selectively recognize and stabilize G4s. TMPyP4, a representative G4 ligand, has been reported to inhibit both RNA polymerase and telomerase activities, thereby suppressing transcription and telomere elongation, respectively.^25,26^ In addition, the binding of TMPyP4 to G4 formed by the *C9orf72* repeat sequence was shown to inhibit the interaction between RBPs and C9orf72 G4, thereby suppressing repeat-associated non-AUG (RAN) translation.^27^ These findings suggest that G4 ligands could serve as effective tools to counteract RBP sequestration. For cellular applications of G4 ligands, structure-selectivity is essential^28^ since the majority of nucleic acids within cells fold into canonical duplexes. Consequently, the development of structure-selective G4 ligands has attracted increasing attention. Among them, pyridostatin (PDS) and PhenDC3 exhibit high affinity and selectivity toward G4 structures while showing minimal binding to duplex DNA.^29,30^ These compounds have been shown to regulate biological processes such as transcription and telomere elongation by stabilizing G4 structures. Therefore, in this study, we investigated the effects of G4 ligands on LLPS induced by RNA G4 (RG4) and a cationic model peptide, which is derived from the RGG domain of FMRP.^24^ It was found that G4 ligands formed aggregates with their target RG4 and further excluded the cationic peptides from these aggregates. However, a more systematic investigation with a series of nucleic acid ligands showed that aggregate formation by G4-ligands depended on their structure-selectivity. These findings provide the first evidence that the structure-selectivity of G4 ligands plays a critical role in controlling LLPS and aggregates of RG4s.

## Results and discussion

### G4-LLPS model system

We used FMR1-RNA derived from the FMR1 mRNA repeat as a model G4-forming RNA (Table 1). G4 formation of FMR1-RNA was monitored by UV-melting curves (Fig. S1A), circular dichroism (CD) measurements (Fig. S1B), and thermal difference spectrum (TDS) (Fig. S1C).^31^ In a buffer containing either 100 mM KCl or 100 mM LiCl, the absorbance of 10 µM FMR1-RNA at 295 nm exhibited a hypochromic transition with increasing temperature in the KCl buffer, indicating a G4 structure.^32^ The CD spectrum of 10 µM FMR1-RNA showed a positive peak at 260 nm and a negative peak at 240 nm, confirming that FMR1-RNA folds into a parallel G4.^24^ The absorbance TDS of a G-quadruplex exhibits a negative peak around 295 nm and two positive peaks around 275 nm and 243 nm.^31^ The two spectra obtained in the KCl buffer and the LiCl buffer were similar; however, the TDS in the KCl buffer exhibited a negative peak at 295 nm and larger positive peaks near 275 nm and 250 nm. A previous study has reported that these differences suggest the formation of G4 in the KCl buffer.^33^ Therefore, the TDS results also support the G4 formation of FMR1-RNA in the KCl buffer but not in the LiCl buffer. We then measured the CD melting curves of FMR1-RNA at 260 nm with different RNA concentrations ranging from 10 µM to 200 µM (Fig. S1D). The melting temperatures calculated from the melting curves are shown in Fig. S1E. It was found that the melting temperature depends on the RNA concentration, suggesting that at least some of the RNA strands form intermolecular G4s. These results are consistent with previous studies showing that parallel G4s tend to dimerize through a head-to-head stacking interaction between G4 units.^34–37^

**Table 1:** RNA and peptide sequences used in this study

	Abbreviation	Sequence
RNAs	FMR1-RNA	GGCGGCGGCGGCGGC
	C9orf72-RNA	GGGGCCGGGGCCGGGGCCGGGGCC
	ds-RNA	UGCGAUAUCGCA
	ss-RNA	UUUUUUUUUUUU
Peptide	RGG-peptide	RRGDGRRRGGGGRGQGGRGRGGGFKGNDDHSRGGW

We reported previously that FMR1-RNA induces LLPS with a cationic peptide.^24^ RGG-peptide, derived from the RGG domain of FMRP (Table 1), which is a typical G4-RBP,^38^ was used as a partner peptide to induce LLPS with FMR1-RNA G4. The net charge of the RGG-peptide is +7. Microscopic observations confirmed that the mixture of 10 µM FMR1-RNA and 10 µM RGG-peptide in the KCl buffer formed droplets *via* LLPS (Fig. S2A left panel), as reported previously.^24^ In contrast, droplet formation was scarcely observed in the LiCl buffer (Fig. S2A right panel), which does not stabilize G4,^39^ indicating that LLPS induced by the RGG-peptide and FMR1-RNA is promoted upon G4 formation. In addition, we used KGG-peptide as a non-G4RBP peptide for control experiments. We reported previously that the KGG-peptide, in which all Arg residues in the RGG-peptide were replaced with Lys residues, does not induce the G4-LLPS.^24^ As expected, the KGG-peptide did not induce LLPS of FMR1-RNA even in the KCl buffer (Fig. S2B). These results confirm that the G4 of FMR1-RNA and the RGG-peptide are essential for the induction of LLPS. Although the LLPS of FMR1-RNA and the RGG-peptide was monitored at 350 nm in our previous report,^24^ here we observed turbidity at 900 nm to avoid overlap with the absorption of a series of small compounds shown later. As expected, it was observed that turbidity at 900 nm increased with reaction time after mixing FMR1-RNA and the RGG-peptide (Fig. S2C).

### Effects of G4 ligands on LLPS

**Fig. 1:**
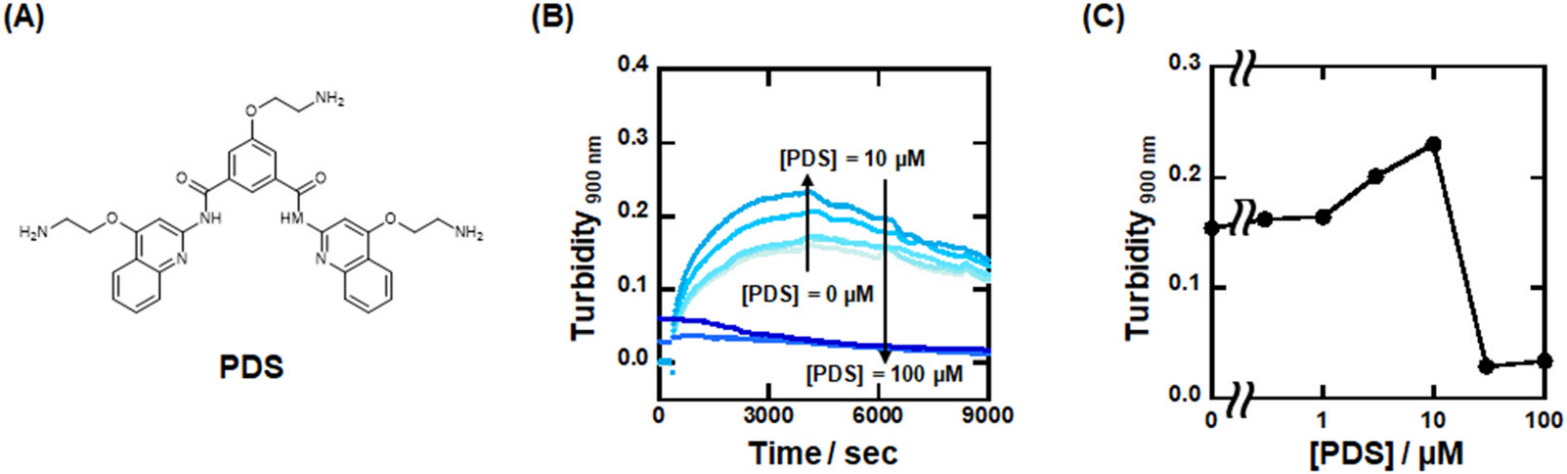
(A) Chemical structure of PDS. (B) Turbidity changes at 900 nm for a mixture of 10 µM FMR1-RNA and 10 µM F-RGG-peptide with various concentrations of PDS (0, 0.3, 1, 3, 10, 30 and 100 µM) in a buffer containing 100 mM KCl and 50 mM MES–LiOH (pH 7.0) at 25 °C. (C) Plots of turbidity at 900 nm for a mixture of 10 µM FMR1-RNA and 10 µM RGG-peptide in the presence of various concentrations of PDS after incubation for 3600 s.

To evaluate the effects of G4 ligands on LLPS induced by FMR1-RNA and the RGG-peptide, we observed the turbidity at 900 nm with PDS (Fig. 1A), which is a representative G4 ligand. PDS was reported to bind and stabilize the telomeric G4 but had no effect on and did not bind to a DNA duplex.^29^ We evaluated the binding affinity of PDS for the FMR1-RNA G4 using FAM-labelled FMR1-RNA (F-FMR1-RNA).^40^ Fig. S3A shows the fluorescence spectra of 50 nM F-FMR1-RNA with various concentrations of PDS. As the fluorescence intensity of F-FMR1-RNA was decreased with increasing concentrations of PDS, we plotted the normalized fluorescence intensity of F-FMR1-RNA at 520 nm for the concentration of PDS in Fig. S3B. The dissociation constant (*K*_d_) of PDS for FMR1-RNA was 0.48 µM at 25 °C.

Once the binding affinity of PDS for FMR1-RNA was confirmed, we evaluated the effect of PDS on LLPS of the FMR1-RNA and RGG-peptide mixture. Fig. 1B shows the turbidity observed at 900 nm of the mixture of 10 µM FMR1-RNA and 10 µM RGG-peptide in the presence of various concentrations of PDS. The turbidity of the mixture at 3600 s, where the maximum turbidity was exhibited, was plotted as a function of PDS concentration (Fig. 1C). The turbidity increased from 0 to 10 µM PDS and then decreased from 10 to 100 µM PDS. The increase and decrease of turbidity with increasing concentration of ligands have been reported for Congo red and bis-ANS,^41^ which were shown to promote LLPS induced by TDP-43 at lower concentrations and suppress it at higher concentrations. The reversible formation and dissolution of the droplets is attributed to electrostatic interactions between the small molecules and the proteins.

**Fig. 2:**
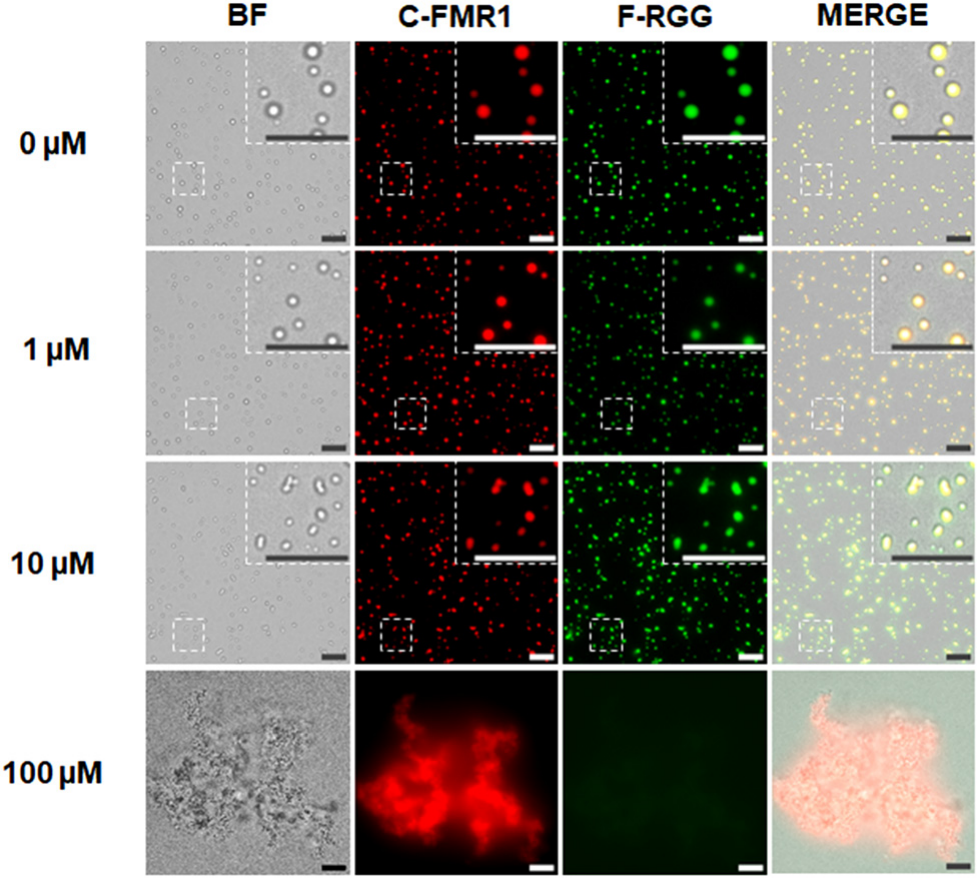
Fluorescence microscopy images showing a mixture of 10 µM C-FMR1-RNA and 10 µM F-RGG-peptide with various concentrations of PDS (0, 1, 10 and 100 µM) after incubation for 3600 s at room temperature. Scale bar = 10 µm. The images of the aggregates were processed with enhanced contrast to improve the clarity of the aggregates.

To study whether the changes in turbidity are due to the promotion and suppression of LLPS by PDS, we observed the self-assembled structures using fluorescence microscopy. Fig. 2 shows fluorescence microscopy images for the mixture of 10 µM Cy5-labelled FMR1-RNA (C-FMR1-RNA) and the 10 µM FAM-labelled RGG-peptide (F-RGG-peptide) with various concentrations of PDS in the KCl buffer. Both C-FMR1-RNA and the F-RGG-peptide were co-localized within the droplets upon addition of 0 to 10 µM PDS. In contrast, when 100 µM PDS was added, aggregate-like structures were observed, suggesting that the decrease of turbidity at higher PDS concentrations could be due to the precipitation of aggregates.

In addition to turbidity, we measured dynamic light scattering (DLS) using unlabelled FMR1-RNA and the RGG-peptide (Fig. S4). In the absence of PDS, a peak was observed at a particle size of 340 nm. As the PDS concentration increased to 1 µM and 10 µM, the peak shifted to 530 nm and 620 nm, respectively. These changes in the particle size are generally consistent with the results of turbidity measurements and microscopic observations. Furthermore, in the presence of 100 µM PDS, the peak position shifted further to 1300 nm. Although particles observed were smaller compared to the aggregates seen under the microscope (Fig. 2), it is possible that larger aggregates were not detected by DLS because they became insoluble and precipitated.

**Fig. 3:**
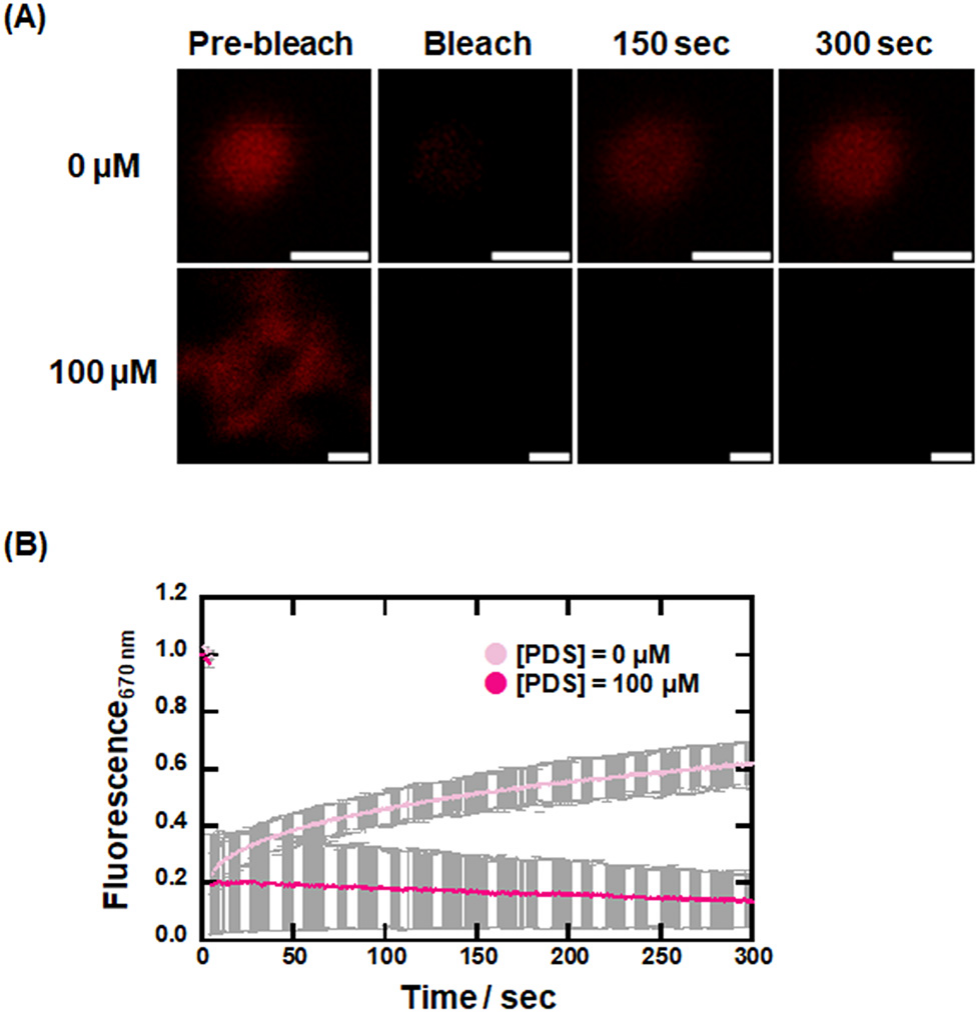
(A) Fluorescence recovery after photobleaching of a droplet of 10 µM C-FMR1-RNA and 10 µM RGG-peptide in the absence and presence of 100 µM PDS at room temperature. Scale bar = 2 µm. (B) FRAP curves for a mixture of 10 µM C-FMR1-RNA and 10 µM RGG-peptide in the absence (pale pink) and presence (pink) of 100 µM PDS.

C-FMR1-RNA fluorescence was observed within both droplets and aggregate-like assemblies regardless of the PDS concentration as shown in the fluorescence microscopy images in Fig. 2. In contrast, fluorescence derived from the F-RGG-peptide became undetectable with increasing PDS concentration, suggesting that the RGG-peptide is excluded from the aggregate-like assembly that was induced by high concentrations of PDS. We will discuss this point later, along with more direct experimental results. To assess the fluidity of these assemblies, fluorescence recovery after photobleaching (FRAP) was performed. Fig. 3A shows the time-lapse images of 10 µM C-FMR1-RNA and 10 µM RGG-peptide in the absence (top) and presence (bottom) of 100 µM PDS. Image acquisition was initiated 30 min after mixing C-FMR1-RNA and RGG-peptide, and photobleaching was performed 5 s after the start of image acquisition. In the absence of PDS, the fluorescence gradually recovered and reached approximately 60% recovery yield after 300 s (Fig. 3B), supporting that C-FMR1-RNA and the RGG-peptide form liquid-like droplets. In contrast, no fluorescence recovery was observed after photobleaching in the presence of 100 µM PDS, demonstrating that PDS induces aggregates of the FMR1-RNA and RGG-peptide mixture. To confirm that droplet formation and aggregate formation are reversible and irreversible, respectively, ATP and 1,6-hexanediol were added one hour after the mixing of FMR1-RNA and the RGG-peptide (Fig. S5). It was found that the addition of 1,6-hexanediol showed no effect on the droplets, whereas the addition of ATP led to the dissolution of the droplets, indicating that droplet formation is reversible and electrostatic interactions between FMR1-RNA and the RGG-peptide play an important role to undergo LLPS. On the other hand, the aggregates of FMR1-RNA and PDS did not redissolve upon the addition of either ATP or 1,6-hexanediol, showing that aggregate formation is irreversible. These effects of the hydrotropes are consistent with the results of the FRAP measurements.

To confirm which molecule, FMR1-RNA or the RGG-peptide, induced the aggregates with PDS, we observed turbidity changes of a mixture of 10 µM FMR1-RNA or 10 µM RGG-peptide with 100 µM PDS (Fig. S6A). The mixture of 10 µM RGG-peptide and 100 µM PDS did not show turbidity changes at 900 nm. In contrast, the mixture of 10 µM FMR1-RNA and 100 µM PDS exhibited a slight increase in turbidity at 900 nm immediately after mixing. To confirm whether this slight increase in turbidity was attributable to aggregate formation, microscopy images of the mixtures were observed. The mixture of FMR1-RNA and PDS was confirmed to form aggregates immediately after mixing (Fig. S6B top). On the other hand, aggregates were not formed by the mixture of the RGG-peptide and PDS (Fig. S6B bottom), indicating that the slight turbidity increase was indeed due to aggregate formation. These observations of subtle turbidity elevation and aggregate formation are consistent with the results shown in Fig. 1B. Collectively, these findings demonstrate that PDS forms aggregates with FMR1-RNA independently of the presence of the RGG-peptide, and further suggest that PDS interferes with the interaction between FMR1-RNA and RGG-peptide. Fig. S6C shows the size distribution of 10 µM FMR1-RNA and 100 µM PDS measured by DLS, showing a particle size of around 960 nm. This suggests that FMR1-RNA and PDS form larger aggregates than droplets of FMR1-RNA and the RGG-peptide. As mentioned above, larger aggregates than these may become insoluble. Furthermore, for determination of the concentration of PDS required to induce aggregation, we investigated the effect of PDS on the aggregates by varying the concentrations of FMR1-RNA G4 and the RGG-peptide (Fig. S7A). The results showed that, in the presence of lower concentrations of FMR1-RNA G4 and the RGG-peptide, the lower concentrations of PDS were required to displace the RGG-peptide and induce aggregate formation. In this experimental system, relatively high concentrations of PDS were required for the exclusion of the RGG-peptide and the aggregation of FMR1-RNA G4; however, the results shown in the phase diagram (Fig. S7B) suggest that the exclusion of the RGG-peptide and the aggregation of FMR1-RNA G4 can be achieved with lower concentrations of the G4 ligand, depending on the concentration of FMR1-RNA and the RGG-peptide.

To investigate whether the aggregate formation induced by PDS is also applicable to other G4-forming sequences, turbidity measurements and microscopy observations were performed using C9orf72-RNA (Table 1), which is derived from the GGGGCC repeat in the *C9orf72* gene. Our previous study demonstrated that C9orf72-RNA also induces LLPS with the RGG-peptide.^24^ Fig. S8A shows the turbidity at various concentrations of PDS. Similar to the results obtained for FMR1-RNA, the turbidity increased up to 10 µM PDS and decreased at higher concentrations (Fig. S8B). Fig. S8C shows microscopy images of mixtures containing 10 µM C9orf72-RNA and 10 µM RGG-peptide with 0, 1, 10, and 100 µM PDS. The formation of aggregates was observed at 100 µM PDS. These results suggest that PDS can form aggregates with other RG4s as well as FMR1-RNA.

In addition to PDS, we used other G4 ligands, PhenDC3,^42^ TMPyP4,^26^ and Thioflavin T (ThT),^43^ for LLPS experiments (Fig. S3, left column). The binding affinity of these ligands for FMR1-RNA was evaluated using F-FMR1-RNA. Fig. S3C, E and G show the fluorescence spectra of 50 nM F-FMR1-RNA with various concentrations of PhenDC3, TMPyP4, or ThT, respectively. The *K*_d_ values of these ligands were evaluated (Fig. S3D, F, H) and are listed in Table S1 with that of PDS. Fig. S9A shows the turbidity for a mixture of 10 µM FMR1-RNA and 10 µM RGG-peptide with various concentrations (0, 0.3, 1, 3, 10, 30 and 100 µM) of PhenDC3 observed at 900 nm. PhenDC3 exhibited effects on LLPS similar to those of PDS, with the turbidity at 900 nm increasing from 0 to 10 µM and decreasing from 10 to 100 µM (Fig. S9A and B). Microscopy observations demonstrated that aggregates were formed upon the addition of 100 µM PhenDC3 (Fig. S9C). At 100 µM PhenDC3, no distinct C-FMR1-RNA fluorescence image was observed, while the bright field (BF) image showed aggregates, suggesting that the fluorescence from C-FMR1-RNA was quenched by PhenDC3. In fact, the fluorescence from C-FMR1-RNA was quenched through the interaction between PhenDC3 and C-FMR1-RNA (Fig. S10). The absence of detected C-FMR1-RNA fluorescence in the solution is consistent with RNA being incorporated into the aggregates, where proximity between PhenDC3 and C-FMR1-RNA leads to fluorescence quenching. TMPyP4 also formed aggregates at 100 µM (Fig. S11). In the BF images, aggregate formation was observed at 100 µM TMPyP4, whereas strong fluorescence from C-FMR1-RNA and the F-RGG-peptide was not detected under the same conditions. Notably, in the F-RGG-peptide fluorescence image, fluorescence from the F-RGG-peptide within the aggregates was completely quenched, which is likely attributable to fluorescence quenching by TMPyP4 localized in the aggregates. The F-RGG-peptide exhibited fluorescence outside the aggregates, indicating that the aggregates formed by TMPyP4 excluded the RGG-peptide. ThT, a structure-selective fluorescent probe for G4, did not induce aggregate formation despite its cationic charge, even at high concentrations (Fig. S12). The G4 ligands must interfere with the interaction between FMR1-RNA and the RGG-peptide for G4 ligands to form G4–G4 ligand aggregation. To confirm this point, we examined the effect of G4 ligand addition on the binding affinity between FMR1-RNA and the RGG-peptide. Fig. S13A shows the fluorescence spectra of the F-RGG-peptide with increasing concentrations of FMR1-RNA in the absence (left) or in the presence of 100 µM PDS (middle) or 100 µM ThT (right). As a decrement in the fluorescence intensity of the F-RGG-peptide was observed upon the addition of FMR1-RNA, normalized *F*_520nm_ {(fluorescence intensity at 520 nm)/(fluorescence intensity at 520 nm without FMR1-RNA)}were plotted for the FMR1-RNA concentration (Fig. S13B) and the *K*_d_ values of FMR1-RNA with the RGG-peptide in each condition were evaluated using eqn (2). The *K*_d_ value of FMR1-RNA with the F-RGG-peptide in the absence of the G4 ligand was 0.08 µM (Fig. S13C) at 25 °C, indicating strong binding affinity. This *K*_d_ increased to 3.0 µM in the presence of 100 µM PDS at 25 °C (Fig. S13C), demonstrating that PDS strongly inhibits the binding between FMR1-RNA and the RGG-peptide. In contrast, upon the addition of ThT, which did not induce aggregate formation, the *K*_d_ value of FMR1-RNA with the RGG-peptide was 0.09 µM (Fig. S13C), showing that 100 µM ThT does not affect the binding. This low inhibitory effect is likely attributable to the weak binding affinity of ThT for FMR1-RNA (Table S1). Collectively, these findings suggest that the binding affinity of ligands with RG4 structures is an important factor for the G4-ligand-induced aggregate formation with FMR1-RNA G4.

### Exclusion of RGG-peptides by G4 ligands

**Fig. 4:**
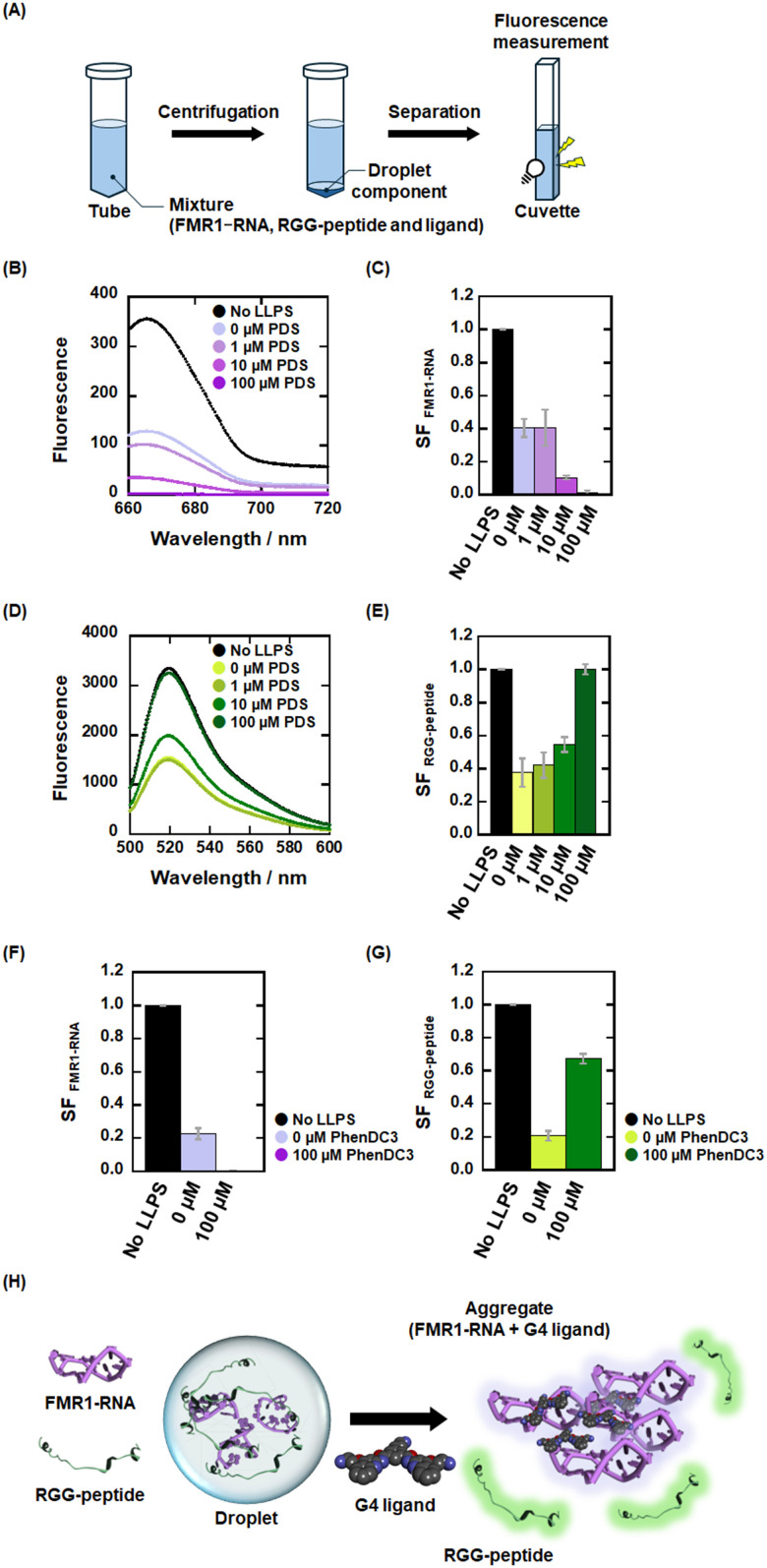
(A) Schematic illustration of the method used to quantify biomolecules outside the droplets or aggregates. RNA, the RGG-peptide, and the ligand were mixed and incubated for 7200 s, followed by centrifugation to collect the supernatant-containing molecules located outside the droplets or aggregates. The amounts of RNA and the RGG-peptide in the supernatant were analyzed by fluorescence measurements. (B) and (D) Fluorescence spectra of C-FMR1-RNA (B) and the F-RGG-peptide (D) in the absence or presence of 100 µM PDS in the buffer consisting of 100 mM KCl and 50 mM MES–LiOH (pH 7.0). The excitation wavelength was 650 nm for C-FMR1-RNA and 490 nm for the F-RGG-peptide. (C) and (E) Bar graphs of supernatant fractions (SF) for C-FMR1-RNA (C) and the F-RGG-peptide (E) for those not undergoing LLPS in the presence of various concentrations of PDS. (F) and (G) Bar graphs of SF for C-FMR1-RNA (F) and the F-RGG-peptide (G) for those not undergoing LLPS in the presence of various concentrations of PhenDC3. (H) Schematic illustration of the exclusion of the RGG-peptide from the aggregation by G4 ligands.

As shown in Fig. 1, it was suggested that PDS induced the aggregate with FMR1-RNA, which leads to the exclusion of the RGG-peptide. To validate RGG-peptide exclusion upon aggregate formation for FMR1-RNA and PDS, we attempted to quantify the distribution of the RGG-peptide inside and outside the self-assemblies. Fig. 4A shows a schematic diagram of the experimental setup. Mixtures of 10 µM C-FMR1-RNA and 10 µM F-RGG-peptide in the absence or presence of 100 µM ligands were prepared and incubated for 2 hours. After the 2-hour incubation period, peptides present outside the assemblies were quantified by centrifuging (15 000 rpm for 15 min) the mixture solution. Fig. 4B shows the fluorescence spectra of 10 µM C-FMR1-RNA with 10 µM RGG-peptide in the absence or in the presence of 0, 1, 10 or 100 µM PDS. In addition to these conditions, the fluorescence spectrum of 10 µM C-FMR1-RNA without 10 µM RGG-peptide was measured as the no-LLPS condition (black in Fig. 4B), which corresponds to the condition where all RNA exists in the solution. The fluorescence intensity for C-FMR1-RNA remaining in the supernatant (outside the droplets or the aggregates) was measured. We evaluated SF_FMR1-RNA_ against the fluorescence intensity under the no-LLPS condition at 670 nm (eqn (3)), where C-FMR1-RNA showed the maximum fluorescence intensity. At 0 and 1 µM of PDS, under the conditions where LLPS occurred, the fluorescence intensity for C-FMR1-RNA was markedly reduced compared to the no-LLPS condition (SF_FMR1-RNA_ = 0.40 and 0.41, respectively) (Fig. 4C), indicating that the majority of the components, C-FMR1-RNA, were sequestered within the droplets upon LLPS formation. At 10 and 100 µM PDS, fluorescence from C-FMR1-RNA significantly decreased (SF_FMR1-RNA_ = 0.10 and 0.01, respectively). This pronounced decrease in fluorescence indicates that the majority of C-FMR1-RNA was incorporated into the aggregates. Next, the fluorescence intensity for the F-RGG-peptide was quantified. Under experimental conditions identical to those used for the C-FMR1-RNA fluorescence measurements, the fluorescence intensity for 10 µM FMR1-RNA and 10 µM F-RGG-peptide in the absence or presence of 0, 1, 10 or 100 µM PDS (Fig. 4D) was measured. The fluorescence intensity of the F-RGG-peptide in the absence of FMR1-RNA was measured as the no-LLPS condition (black in Fig. 4D). The SF_RGG-peptide_ with respect to the F-RGG fluorescence intensity under the no-LLPS condition was also calculated using eqn (3). Under the LLPS-inducing conditions without PDS, the fluorescence intensity of the F-RGG-peptide decreased similar to that of C-FMR1-RNA (SF_RGG-peptide_ = 0.38), indicating that the RGG-peptide was incorporated into the droplets (Fig. 4E). However, upon the addition of 1, 10, or 100 µM PDS, the fluorescence intensity of the F-RGG-peptide increased to a level comparable to that observed under the no-LLPS condition (SF_RGG-peptide_ = 0.42, 0.55, 1.00, Fig. 4E). Although further verification using other methods, such as microequilibrium dialysis, are required, these results demonstrated that the F-RGG-peptide was excluded from the aggregates, in contrast to C-FMR1-RNA, which is included in the aggregates.

In addition to PDS, the exclusion capability of PhenDC3 was evaluated. Fig. S14A and B show the fluorescence spectra of 10 µM C-FMR1-RNA and 10 µM F-RGG-peptide, respectively, in the absence or presence of 100 µM PhenDC3. The experimental conditions were identical to those used in the experiment with PDS as shown in Fig. 4B and D. The fluorescence intensities of C-FMR1-RNA remaining in the supernatant (outside the droplets or the aggregates) were measured. Similar to PDS, at 100 µM PhenDC3, the fluorescence intensity of C-FMR1-RNA was significantly reduced compared to the no-LLPS condition (relative *F*_670_ = 0.002, Fig. 4F), indicating that the aggregation triggered by PhenDC3 also included the C-FMR1-RNA aggregate. In contrast, the fluorescence intensity of the F-RGG-peptide increased compared to the fluorescence intensity without the F-RGG-peptide (relative *F*_520_ = 0.67, Fig. 4G). Although the fluorescence increase was lower than that observed with PDS (Fig. 4E), PhenDC3 also demonstrated the exclusion of the F-RGG-peptide from the aggregates at high concentrations. These results indicate that the G4 ligand–FMR1-RNA aggregates are capable of excluding RNA-binding peptides/proteins. Thus, it may be possible to induce the formation of aggregates that do not incorporate RBPs by employing such a strategy (Fig. 4H).

### Structure-selectivity of ligands for inducing aggregate formation

**Fig. 5:**
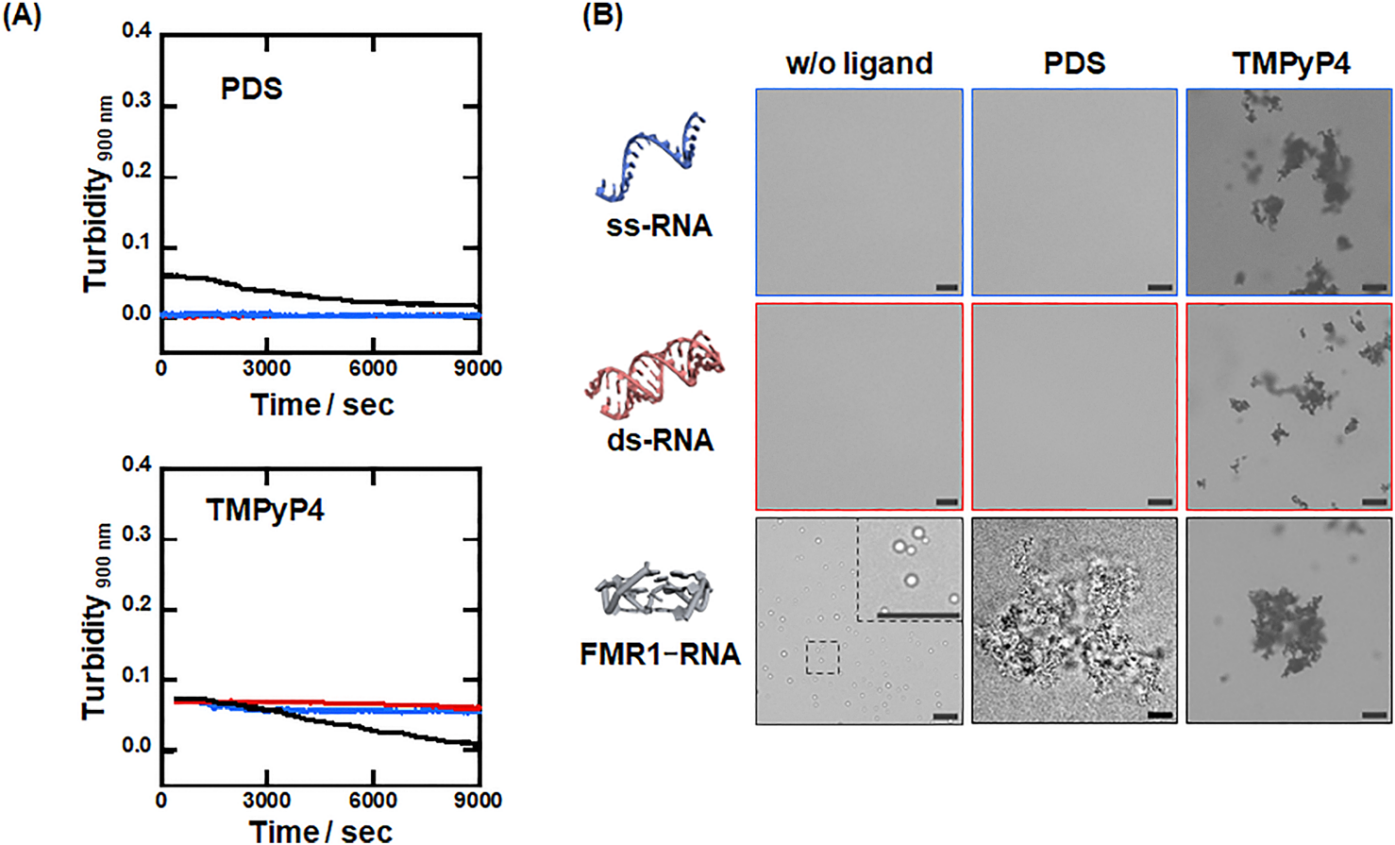
(A) Turbidity changes at 900 nm for a mixture of 10 µM RNAs (ss-RNA, ds-RNA and FMR1-RNA) and 10 µM RGG-peptide in the absence or presence of 100 µM PDS (top) and TMPyP4 (bottom) in a buffer containing 100 mM KCl and 50 mM MES–LiOH (pH 7.0) at 25 °C. (B) Microscopy images showing mixtures of 10 µM RNAs (ss-RNA, ds-RNA and FMR1-RNA) and 10 µM RGG-peptide in the absence or presence of 100 µM PDS or TMPyP4 after incubation for 3600 s at room temperature. Scale bar = 10 µm. The images of the aggregates were processed with enhanced contrast to facilitate visualization of the aggregates.

To therapeutically exploit the ability of G4 ligands to form aggregates with FMR1-RNA and to exclude the RGG-peptide, it is essential that this aggregation property is expressed in a structure-selective manner. To investigate the structure-selectivity of G4 ligands for aggregate formation, turbidity measurements and microscopy observations of the mixture of RNAs, RGG-peptide, and ligands were conducted using single-stranded RNA (ss-RNA, Table 1) and double-stranded RNA (ds-RNA, Table 1) as well as FMR1-RNA G4. In addition to PDS, we used TMPyP4 due to its non-structure-selective binding against nucleic acid secondary structures.^28^Fig. 5A shows the turbidity for mixtures of 10 µM RNAs (ss-RNA, ds-RNA, or FMR1-RNA) and the 10 µM RGG-peptide with 0 or 100 µM of PDS and TMPyP4 at 900 nm. The mixtures of the RGG-peptide and either ss-RNA or ds-RNA showed no increase in turbidity at 900 nm, indicating that the RGG-peptide does not undergo LLPS with ss-RNA or ds-RNA. These results offer further support that only G4 induces LLPS with the RGG-peptide as shown previously. Microscopy images for the mixtures of 10 µM RNAs and 10 µM RGG-peptide in the presence of 100 µM PDS are shown in Fig. 5B. Aggregation was observed for the mixture of 10 µM FMR1-RNA, but not in the mixtures of ss-RNA or ds-RNA. These results suggest that the aggregation of PDS occurred with FMR1-RNA G4 but not with RNA folded into other secondary structures. PDS has been reported to be a structure-selective G4 ligand, and its structure-selective aggregate formation is considered to arise from this specific binding property toward nucleic acids. At 100 µM TMPyP4, a slight increase in turbidity was observed for all RNA species in the absence of the RGG-peptide, suggesting that aggregates were formed (Fig. 5A bottom). Microscopy observations confirmed that TMPyP4 induced aggregates with ss-RNA, ds-RNA and FMR1-RNA, indicating that TMPyP4 forms aggregates in a structure-non-selective manner. Since TMPyP4 binds to RNAs in a non-structure-selective manner,^28^ its lack of binding selectivity is likely reflected in this non-selective aggregate formation. The structure-selective aggregate of FMR1-RNA induced by PDS and the non-structure-selective aggregate induced by TMPyP4 are likely attributable to differences in the interactions that induce aggregation. Because TMPyP4 possesses not only a large planar structure but also strongly positively charged functional groups, it may form electrostatic interactions with RNA G4 more readily than PDS. The aggregate formation was examined under different ionic strength conditions to investigate the mode of interactions responsible for inducing the aggregation. Fig. S15 shows microscopy images of mixtures of 10 µM FMR1-RNA and RGG-peptide in the absence or presence of 100 µM PDS or TMPyP4 at 1, 10, 100 and 1000 mM KCl. Under high-salt conditions, electrostatic interactions are weakened; therefore, aggregates driven by such interactions are expected to disappear. In the absence of G4 ligands, droplet formation was observed at 1, 10 and 100 mM KCl, whereas the droplets disappeared at 1000 mM KCl. PDS formed aggregates under all KCl concentration conditions, suggesting that the primary interaction responsible for PDS-induced aggregation is a non-electrostatic interaction, such as π–π stacking and hydrogen-bonding interactions. These results show that the structure-selectivity of ligands influences which RNA secondary structures form aggregates and indicate that G4 structure-selectivity of ligands is essential for the selective aggregation of G4s. This is important for the sequestration of RNA-binding proteins from droplets involving the target G4 in a selective manner, which could lead to higher drug efficacy with fewer side effects.

Finally, we examined LLPS and LSPS, and the effects of G4 ligands not only for FMR1-RNA, (GGC)_4_ involving one G4 unit, but also for the G4-forming sequences, (GGC)_8_ (FMR1-RNA 8) and (GGC)_16_ (FMR1-RNA 16), which involve two and four G4 units, respectively. To ensure consistent G4 unit concentrations, we set the strand concentrations for each to 10 µM, 5 µM and 2.5 µM, respectively. The left column in Fig. S16 shows the microscopic observations following the addition of 10 µM RGG-peptide and 120 min incubation at room temperature. Compared to FMR1-RNA, a greater number of larger droplets were observed with FMR1-RNA 8 and FMR1-RNA 16. These results suggest that RNA with a higher number of repeats, which is a factor in disease onset, may be capable of inducing LLPS at lower concentrations. Moreover, the effects of PDS on the longer RNAs showed a similar trend to the one on FMR1-RNA. Although further investigations of binding affinity and stoichiometry are required, these results indicate that G4 ligands can exclude peptides from long-chain G4-forming RNAs through a similar mechanism.

## Discussion

Controlling the behaviour of biomolecular condensates (droplets) using small compounds is a rapidly evolving field, often referred to as the development of condensate-modifying therapeutics^44^ with LLPS modulators.^45–47^ In addition to the representative examples shown below, a wide variety of LLPS modulators have been reported. To dissolute pathological condensates, 4,4′-dianilino-1,1′-binaphthyl-5,5′-disulfonic acid (bis-ANS) and related compounds were reported as potent biphasic modulators of LLPS mediated by TDP-43 and other proteins.^41^ Depending on the concentration, bis-ANS can both induce LLPS and prevent LLPS. The biphasic effects on LLPS are similar to the results obtained in this study, suggesting that the ability to promote LLPS at low concentrations and inhibit it at high concentrations may represent the typical small modulators’ effects on biomolecular LLPS. The screening study for the modulation of nucleocapsid protein condensation identified small molecules with anti-coronavirus activity.^48^ Rosmanol quinone (RQ) was discovered to force β-catenin (an oncogenic protein in liver cancer) into cytoplasmic condensates, leading to prevention of β-catenin from entering the nucleus and activating cancer-promoting genes.^49^ Methylene blue was reported to promote tau LLPS and accelerate the liquid-to-gel transition of tau droplets and inhibit fibril formation.^50^ Lipoamide was identified to prevent the formation of stress granules and alleviate pathology in ALS models involving proteins like FUS and TDP-43.^51^ These previous studies indicate that small compounds capable of modulating LLPS hold promise as therapeutic agents for various diseases.

There are fewer examples of LLPS modulators targeting nucleic acids compared to those targeting proteins. It was reported that cisplatin partitions into transcriptional condensates and affects transcription, whereas in the same reports, a CDK7 inhibitor and a BRD4 inhibitor were found to control nucleic acid condensates by disrupting the protein–DNA interaction network that is required for super-enhancer droplets in cancer cells.^52^ Recently, Ginsenoside compound K, a major metabolite of Panax ginseng, was found to inhibit the gelation of GGGGCC repeats both *in vitro* and *in vivo*.^53^ Moreover, this compound reduces the co-aggregation of RNA and arginine-rich poly-dipeptides *via* electrostatic interactions. As for the exclusion of peptides/proteins from assemblies of nucleic acids and peptides/proteins, it was also reported that the hydrogels of CGG-G4RNA and FMRpolyG protein formed substantially smaller droplets after protoporphyrin IX treatment, suggesting that this compound unfolds CGG-G4RNA, which acts as a scaffold for phase transition processes and reducing FMRpolyG binding to CGG-G4RNA and the phase transition.^9^ In the above-mentioned studies, peptides/proteins were successfully excluded from assemblies using compounds that target different RNA G4 structures. The former study proposes that the binding of the compound to RNA G4 inhibits the dimerization and oligomerization of RNA G4, which in turn leads to the exclusion of peptides. In contrast, the latter study suggests that the compound destabilizes the RNA G4, leading to the disassembly of the aggregates. Regarding the exclusion mechanism of peptides and proteins by small compounds, this study found that when the binding between FMR1-RNA G4 and G4 ligands is strong, RNA G4–G4 ligand aggregates were formed. Furthermore, it was shown that G4 ligands exclude RGG peptides from the aggregates by competing with the binding between FMR1-RNA G4 and the peptides. Although the previous studies have not examined the binding affinity of the small compounds to the target G4s or the effects of small-molecule compounds with different binding affinities on LLPS, the results obtained in this study suggest that the effect of G4 ligands on G4 LLPS may vary depending on the binding affinity. The ability to exclude peptides and proteins from aggregates in a manner that is dependent on their binding affinity to the target, as well as being both structure-selective and sequence-selective, as well as RNA-selective, such as carboxy PDS,^54^ is expected to improve efficacy and reduce side effects in drug development. It is thus required to more systematically investigate the effects of G4 ligands with different charges, affinities, and selectivities on G4 LLPS.

## Conclusion

G4 structures have been attracting attention as therapeutic targets for diseases such as cancer, leading to active efforts in the development of G4 ligands. Most of these ligands have been designed to treat diseases by inhibiting enzymatic reactions that suppress the expression of disease-related genes. Here, we demonstrated that G4 ligands can induce the formation of G4–G4 ligand aggregates. Furthermore, structure-selective G4 ligands formed aggregates only with G4 structures, while no aggregation was observed with single-stranded or double-stranded RNAs. Importantly, since these aggregates exclude the RGG-peptide, this aggregation-based approach is expected to prevent the incorporation of RBPs into aggregates. G4s are increasingly being recognized as functional components involved in various LLPS phenomena. In addition to FMRP, G4s have been reported to interact with proteins such as FUS, DNAPTP6, and HMGB1, thereby inducing LLPS,^55–57^ suggesting that the G4-targeting LLPS controlling strategy using G4 ligands could be widely applicable.

## Experimental

### Sample preparation

All oligonucleotides were purchased from Hokkaido System Science Co., Ltd (Hokkaido, Japan). The HPLC grade peptide was purchased from GenScript Japan Inc. (Tokyo, Japan). Peptide concentrations were measured at 280 nm using a UV-1900 spectrophotometer. Thioflavin T (ThT) and TMPyP4 were purchased from FUJIFILM Wako Pure Chemical Corporation (Osaka, Japan). Pyridostatin and PhenDC3 were purchased from Cayman Chemical (Ann Arbor, MI, USA). Extinction coefficients for single-strand FMR1-RNA, C9orf72-RNA, ds-RNA and ss-RNA were calculated from mono and dinucleotide data using the nearest-neighbor approximation model. The stock solutions of RNA were stored at −30 °C. Single-strand concentrations of the RNA were determined by measuring the absorbance at 260 nm at 90 °C. The stock solutions of ligands (1 mM in dimethyl sulfoxide) were stored at −30 °C in the dark. Before all measurements, the RNA in a buffer containing 100 mM KCl and 50 mM 2-(*N*-morpholino) ethanesulfonate (MES)–LiOH (pH 7.0) was heated at 93 °C for 5 min, gently cooled at −0.5 °C min^−1^, and incubated at 25 °C.

### UV melting measurements

The melting curves for the RNA oligonucleotides were measured by monitoring the absorption at 295 nm using a UV spectrophotometer (UV-1900; Shimadzu Corporation, Kyoto, Japan) connected to a temperature controller (Shimadzu Corporation, Kyoto, Japan). RNA samples in a buffer containing 100 mM KCl and 50 mM MES–LiOH (pH 7.0) were heated at a rate of 0.5 °C min^−1^ from 25 to 95 °C to observe the thermal denaturation curves.

### CD spectrum measurements

The circular dichroism (CD) spectra of 10 µM FMR1-RNA were measured in a buffer containing 100 mM KCl and 50 mM MES–LiOH (pH 7.0) at 25 °C using a J-820 spectropolarimeter (JASCO Co. Ltd, Tokyo, Japan) equipped with a JASCO PTC-424L temperature controller using a cuvette with a 0.1 cm path length under a N_2_ gas flow. The spectra were averaged over at least three scans.

### Dynamic light scattering

Dynamic light scattering (DLS) measurements were performed using a Zetasizer Nano-ZA (Malvern Instruments). The measurements were carried out using solutions containing 10 µM RNA oligonucleotide, 10 µM RGG-peptide and PDS at various concentrations (0, 1, 10 and 100 µM) in a UV cuvette microcell. Each sample was measured 10 times with three accumulations per measurement. All measurements were conducted in a buffer containing 100 mM KCl and 50 mM MES–LiOH (pH 7.0) at 25 °C. The RNA and peptide samples were separately heated to 95 °C for 5 min and then gradually cooled to 25 °C at a rate of −0.5 °C min^−1^. Particle size distributions were analyzed by DLS, as described previously.^58^

### UV-absorbance spectra and thermal difference spectra

UV-absorbance spectra measurements were performed using a UV-1800 spectrophotometer (Shimadzu Corporation, Kyoto, Japan) equipped with a temperature controller. The absorbance was monitored for 10 µM FMR1-RNA in the presence of either KCl or LiCl. For each sample, the thermal difference spectrum (TDS) was obtained by subtracting the absorbance spectrum recorded at a low temperature (25 °C) from that at a high temperature (95 °C), as described previously.^31^

### Turbidity measurements

Turbidity was measured at 900 nm using a UV spectrophotometer UV-1900 connected to a temperature controller. The measurements were performed using solutions of 10 µM RNA oligonucleotide, 10 µM RGG-peptide and mixtures of both using a quartz cell with a 1 cm path length. Various concentrations of the RGG-peptide solution (1, 3, 5, 10 or 15 µM) were added to the DNA solution (a final concentration of 10 µM) after 300 s incubation following the initiation of measurement. All measurements were conducted in a buffer containing 100 mM KCl and 50 mM MES–LiOH (pH 7.0) at 25 °C. The DNA and peptide samples were separately heated to 95 °C for 5 min and then gently cooled to 25 °C at a rate of −0.5 °C min^−1^.

### Microscopy measurements

Images were captured using an EVOS M5000 fluorescence microscope (Thermo Fisher Scientific, Waltham, MA, USA) with a 60× oil-immersion objective lens. Samples were imaged in a buffer containing 100 mM KCl and 50 mM MES–LiOH (pH 7.0) after incubation for 30 min at room temperature. Before imaging, RNA and peptide samples were separately heated to 95 °C for 5 min and then gently cooled to 25 °C at a rate of −0.5 °C min^−1^. To measure the droplet area, the fluorescence intensities inside the droplets were analyzed using ImageJ software (National Institutes of Health, Bethesda, MD, USA). The average fluorescence intensity values within each droplet were calculated by averaging these values on a per-droplet basis after subtracting the background.

### Evaluation of the binding affinity of G4 ligands for FMR1-RNA

Fluorescence spectra of F-FMR1-RNA were measured using an FP-8200 spectrofluorometer (JASCO Co. Ltd, Tokyo, Japan) with a 0.3 cm path length quartz cuvette. The fluorescence spectra were measured at an excitation wavelength of 490 nm for F-FMR1-RNA and 649 nm for C-FMR1-RNA. All experiments were performed in a buffer containing 100 mM KCl and 50 mM MES–LiOH (pH 7.0) at 25 °C. Normalized *F*, (fluorescence intensity with the G4 ligand) – (fluorescence intensity without the G4 ligand), was plotted against the concentration of G4 ligands, and fitted with the following equation using KaleidaGraph (Synergy Software, Reading, PA, USA) to evaluate the *K*_d_ value for G4 ligands with FMR1-RNA:1
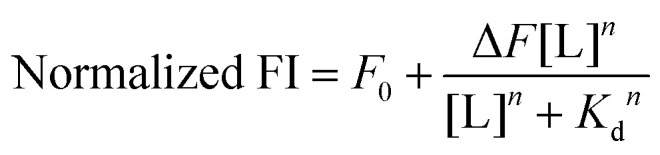
where *F*_0_ is the initial fluorescence intensity, Δ*F* is the change in fluorescence intensity, [L] is the concentration of the G4 ligand, *K*_d_ is the apparent dissociation constant, and *n* is the Hill constant.

### Fluorescence recovery after photobleaching (FRAP)

FRAP experiments were performed for a mixture of 10 µM cyanine5-modified FMR1-RNA and 10 µM RGG-peptide in a buffer containing 100 mM KCl and 50 mM MES–LiOH (pH 7.0) using a confocal microscope (A1R, Nikon Corporation, Tokyo, Japan) with a 60× oil-immersion objective lens. Photobleaching was performed at 640 nm and the fluorescence intensity at the bleached spot was measured using ImageJ.

### Evaluation of the binding affinity between FMR1-RNA and the RGG-peptide

The binding affinity of FMR1-RNA for the RGG-peptide was evaluated by fluorescence spectroscopy using an FP-8200 spectrofluorometer with a 0.3 cm path length quartz cuvette. Fluorescence spectra of the 50 nM F-RGG-peptide were measured from 510 nm to 650 nm. The fluorescence spectra were measured at an excitation wavelength of 495 nm. All experiments were performed in a buffer containing 100 mM KCl and 50 mM MES–LiOH (pH 7.0) at 25 °C. *F*_520nm_ was plotted against the concentration of the FMR1-RNA in the absence or presence of 100 µM PDS or ThT, and fitted with the following equation using KaleidaGraph to evaluate the *K*_d_ for G4 ligands with FMR1-RNA:2
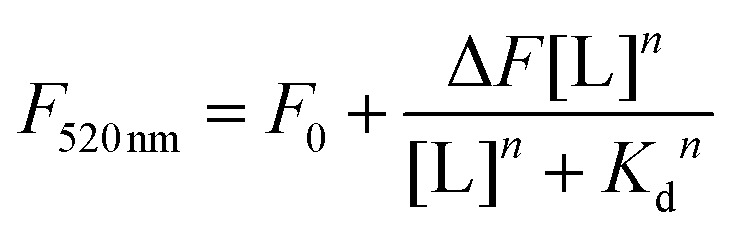
where *F*_520nm_ is the fluorescence intensity at 520 nm, *F* is the fluorescence intensity in the absence of the G4 ligand, Δ*F* is the fluorescence change, [L] is the concentration of the G4 ligand, *K*_d_ is the apparent dissociation constant, and *n* is the Hill constant.

### Quantify the distribution of peptides and RNA inside and outside the self-assemblies

The distributions of C-FMR1-RNA and the F-RGG-peptide inside and outside the droplets and aggregates were quantified by fluorescence spectroscopy. After 2 hours from mixing the 10 µM F-RGG-peptide and 10 µM C-FMR1-RNA, the samples were centrifuged (15 000 rpm; 15 min) to pellet the aggregates. The supernatant was collected for fluorescence measurements in a 0.3 cm cuvette, and the amounts of C-FMR1-RNA and the F-RGG-peptide present in the supernatant were quantified. Fluorescence measurements were performed with excitation wavelengths of 650 nm and 495 nm for C-FMR1-RNA and the F-RGG-peptide, respectively. Relative amounts of C-FMR1-RNA (supernatant fraction for FMR1-RNA: SF_FMR1-RNA_) and the F-RGG-peptide (supernatant fraction for the RGG-peptide: SF_RGG-peptide_) were evaluated from the fluorescence intensity of C-FMR1-RNA and the F-RGG-peptide at 670 nm (*F*_670_) and 520 nm (*F*_520_), respectively, using the following equation:3
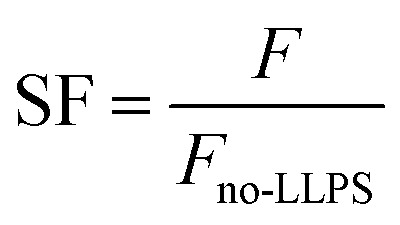
where *F*_no-LLPS_ is the fluorescence intensity of C-FMR1-RNA and the F-RGG peptide under no-LLPS conditions at 670 nm or 520 nm, respectively. SF is normalized *F*_670_ or *F*_520_ against *F*_no-LLPS_ at 670 nm or 520 nm, respectively.

## Author contributions

Conceptualization: D. M.; methodology: D. M.; formal analysis: Y. H., R. S., M. A., N. T. and M. T.; investigation: Y. H., R. S., M. A., N. T. and M. T.; data curation: Y. H., R. S., M. A., N. T. and M. T.; writing—original draft preparation: Y. H. and R. S.; writing—review and editing: K. K. and D. M.; visualization: D. M.; supervision: T. G., K. K. and D. M.; project administration: D. M.; funding acquisition: Y. H., K. K. and D. M.

## Conflicts of interest

There are no conflicts to declare.

## Supplementary Material

CB-007-D6CB00020G-s001

## Data Availability

The data supporting this article have been included as part of the supplementary information (SI). Supplementary information is available. See DOI: https://doi.org/10.1039/d6cb00020g.
