# Supplementary material for: Exclusion of RNA-binding domains from G-quadruplex condensates by G-quadruplex ligands
Source: RSC Chem Biol. 2026 Jul 1;7(8):1578–88. doi: 10.1039/d6cb00020g (PMC13359080; doi:10.1039/d6cb00020g)
Supplement: Supplementary file 1 [file CB-007-D6CB00020G-s001.pdf]

Supplementary Information (SI) for RSC Chemical Biology.
This journal is © The Royal Society of Chemistry 2026

Electronic Supplementary Material (ESI) for XXX.
This journal is RSC Chemical Biology 2026

Supporting Information

Exclusion of RNA-binding domains from G-quadruplex condensates by G-quadruplex ligands

Yoshiki Hashimoto,[a,†] Ryosuke Suzuki,[a,†] Mizuho Aya,[a,†] Nagisa Takamiya,[a] Mitsuki Tsuruta,[a,b] Takeru Torii,[a] Toshiyuki Goto,[c] Keiko Kawauchi,[a] Daisuke Miyoshi[a,*]

[a] Faculty of Frontiers of Innovative Research in Science and Technology (FIRST), Konan University, Chuo-ku, Kobe, 650-0047, Japan
[b] School of Physical and Mathematical Sciences, Nanyang Technological University, Singapore 637371, Singapore
[c] Graduate School of Science, Technology and Innovation, Kobe University, Rokko, Nada-ku, Kobe 657-8501, Japan

† These authors contributed equally
Email: miyoshi@konan-u.ac.jp

Table S1. Dissociation constant (K_d) value at Hill constant (n) of PDS, PhenDC3, TMPyP4 and ThT with FMR1-RNA at 25 °C.

Ligand	K_d (μM)	n
PDS	0.48	1.8
PhenDC3	0.29	1.7
TMPyP4	0.23	1.6
ThT	11	1.0

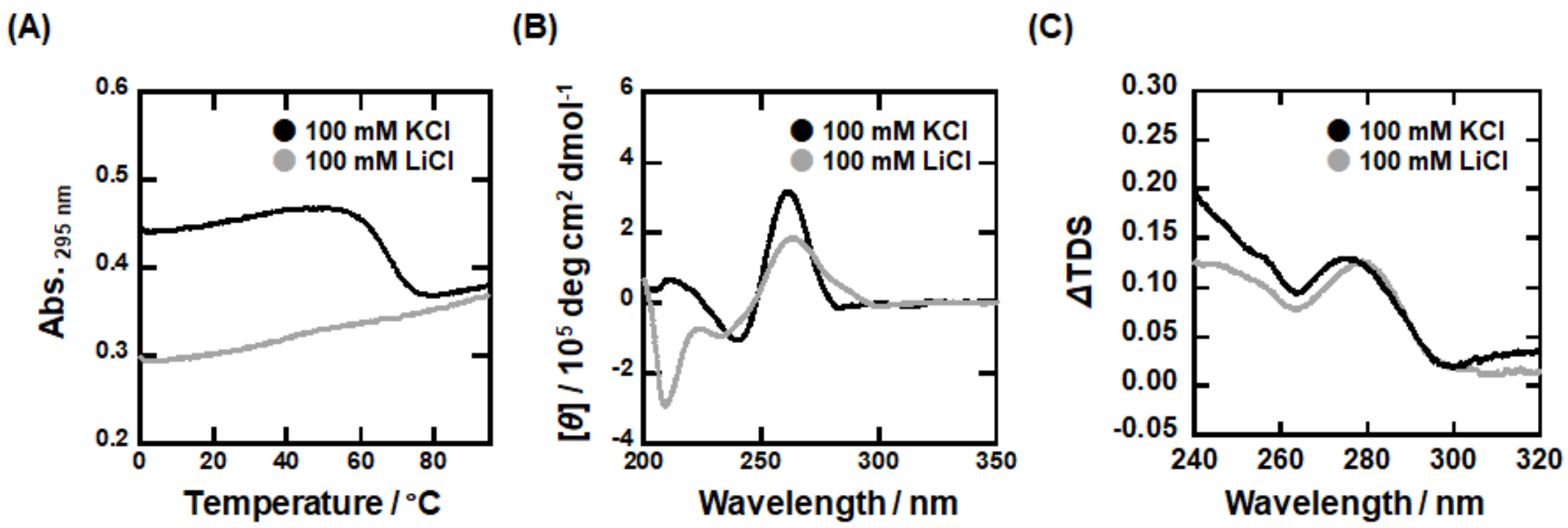

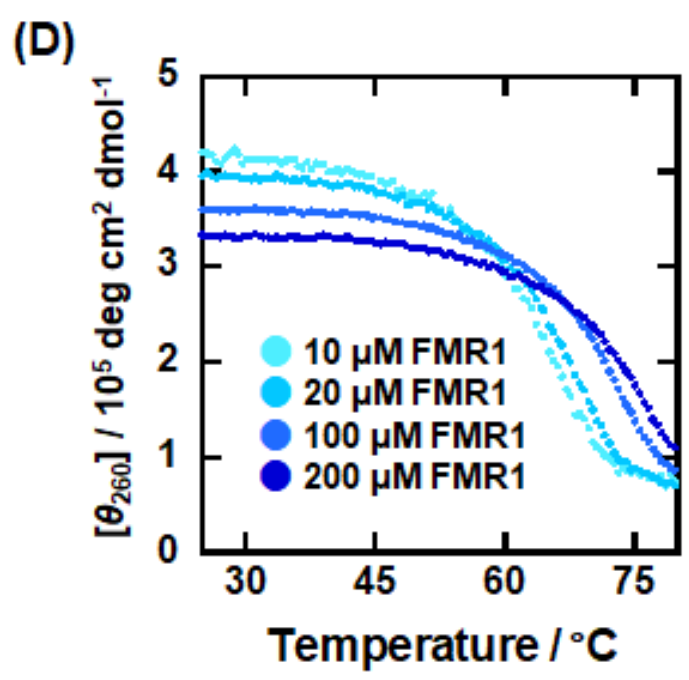

(E)

RNA / µM	T_m
10	69.8
20	72.1
100	~75
200	~75

Figure S1. UV melting curves monitored at 295 nm (A), CD spectra (B), and TDS (C) of FMR1-RNA in a buffer containing 50 mM MES-LiOH (pH 7.0), with either 100 mM KCl (black) or 100 mM LiCl (gray). RNA concentrations were 20 µM for (A, B) and 10 µM for (C). Measurements for (B) and (C) were performed at 25 °C. (D) CD melting curves traced at 260 nm for 10, 20, 100 and 200 µM FMR1 RNA in the buffer containing 100 mM KCl and 50 mM MES-LiOH (pH 7.0). (E) The T_m values evaluated from the melting curves of 10, 20, 100 and 200 µM FMR1 RNA.

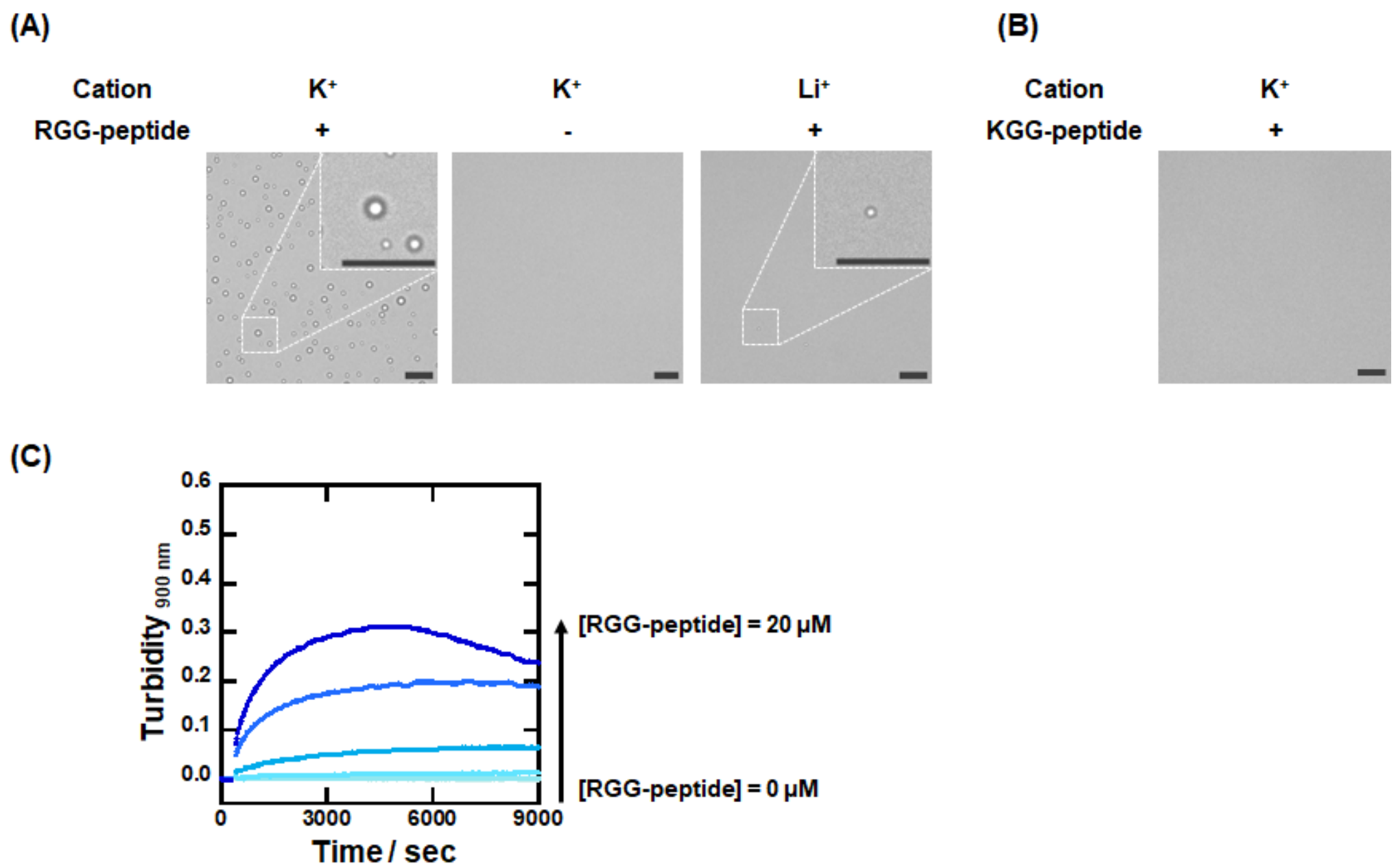

Figure S2. (A) Bright field images of 10 μM FMR1-RNA without and with 10 μM RGG-peptide after incubation for 3600 s at room temperature in the presence of 100 mM KCl or 100 mM LiCl. Scale bar = 10 μm. (B) Bright filed image of a mixture of 10 μM FMR1-RNA with 10 μM KGG-peptide [KKGDGKKKGGGGKGQGGKGKGGGFKGNDDHSKGGW] after incubation for 3600 s at room temperature. (C) Turbidity changes at 900 nm for the mixtures of 10 μM FMR1-RNA with various concentrations of RGG-peptide (0, 1, 2, 5, 10 and 20 μM). All experiments were carried out in a buffer containing 50 mM MES-LiOH (pH 7.0) with 100 mM KCl or LiCl at 25 °C.

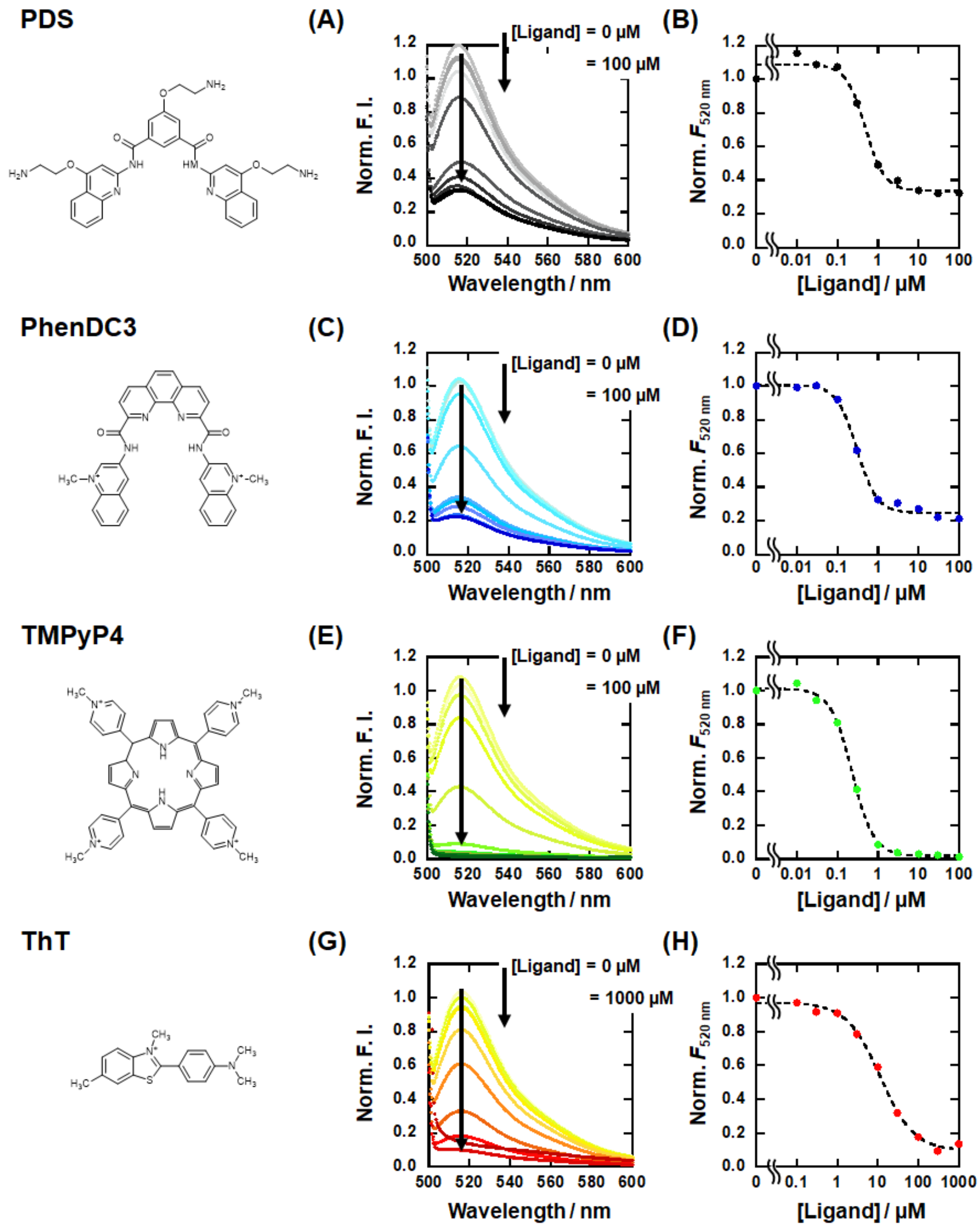

Figure S3. (A, C, E) Fluorescence spectra of 50 nM F-FMR1-RNA in the presence of various concentrations (0, 0.01, 0.03, 0.1, 0.3, 1, 3, 10, 30 and 100 μM) of PDS, PhenDC3, or TMPyP4, and various concentrations (0, 0.1, 0.3, 1, 3, 10, 30, 100, 300 and 1000 μM) of ThT (G). (B, D, F, H) Normalized fluorescence intensity versus concentration of PDS (B), PhenDC3 (D), TMPyP4 (F), or ThT (H). The theoretical equation (Eq-1 in the main text) was used to determine the dissociation constant (K_d) and Hill constant (n) values for the binding of PDS, PhenDC3, TMPyP4 and ThT to F-FMR1-RNA at 25 °C. These values are listed in Table S1. Chemical structures of PDS, PhenDC3, TMPyP4 and ThT are shown on the left. All experiments were carried out in the buffer containing 100 mM KCl and 50 mM MES-LiOH (pH 7.0).

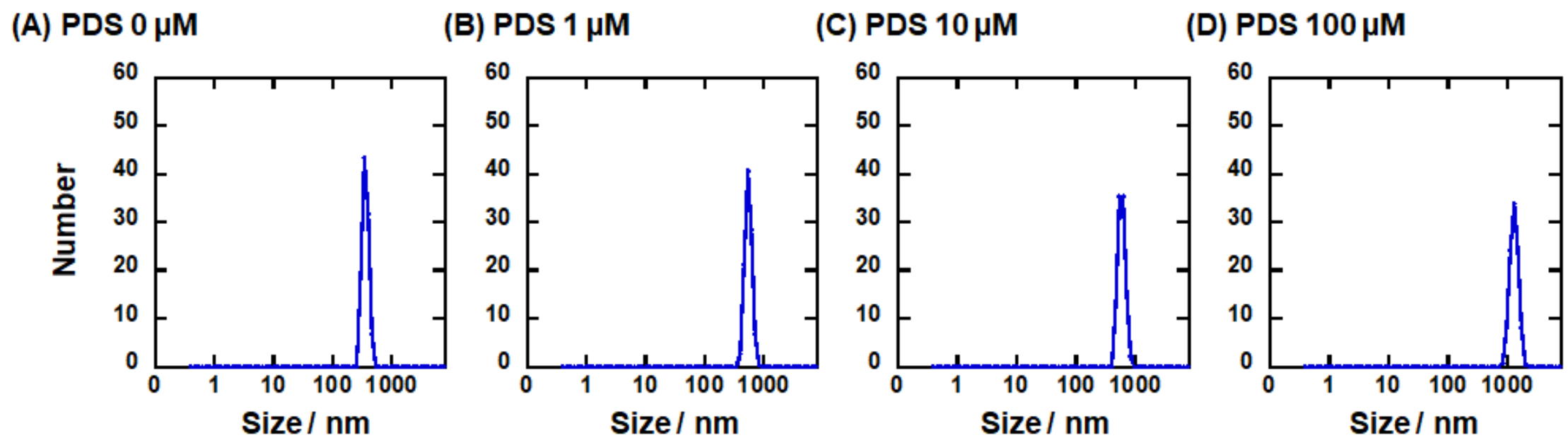

Figure S4. DLS measurements of the mixture of 10 μM FMR1-RNA and 10 μM RGG-peptide in the presence of 0 (A), 1 (B), 10 (C) and 100 μM (D) PDS. All measurements were carried out in the 100 mM KCl buffer at 25°C after 3600 s incubation.

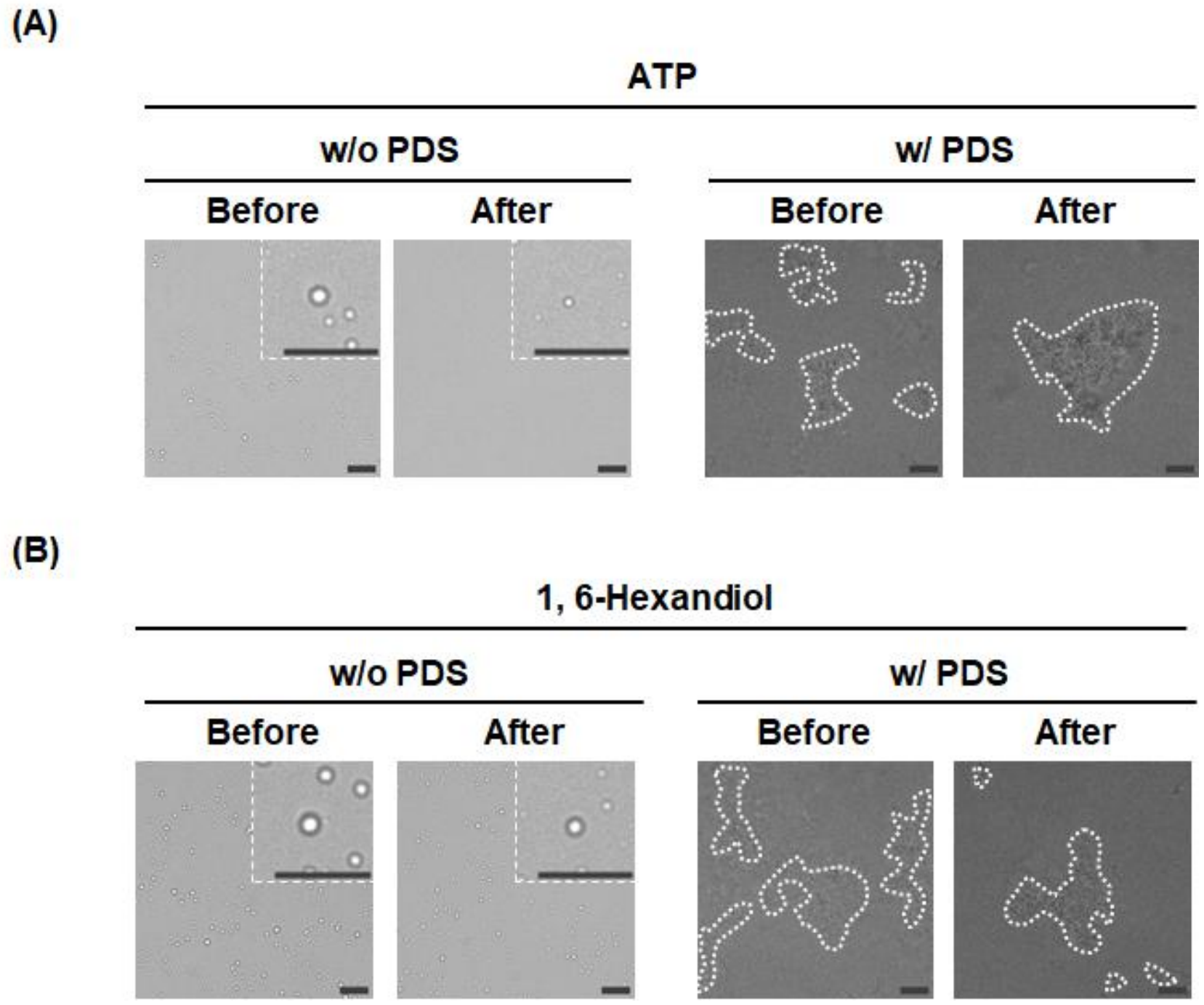

Figure S5. (A) Bright field images of mixtures containing 10 µM FMR1-RNA and 10 µM RGG-peptide in the absence (left) or presence (right) of 100 µM PDS. Images were acquired after incubation for 3600 s and again after the subsequent addition of 10 mM ATP followed by a further 3600 s incubation. (B) Bright field images of mixtures containing 10 µM FMR1-RNA and 10 µM RGG-peptide in the absence (left) or presence (right) of 100 µM PDS. Images were acquired after incubation for 3600 s and again after the subsequent addition of 20 wt% 1,6-hexanediol followed by a further 3600 s incubation. All images were taken in the buffer containing 100 mM KCl and 50 mM MES-LiOH (pH 7.0) at room temperature. Scale bar = 10 µm. The aggregates observed in the presence of 100 µM PDS are outlined with white dashed lines for clarity.

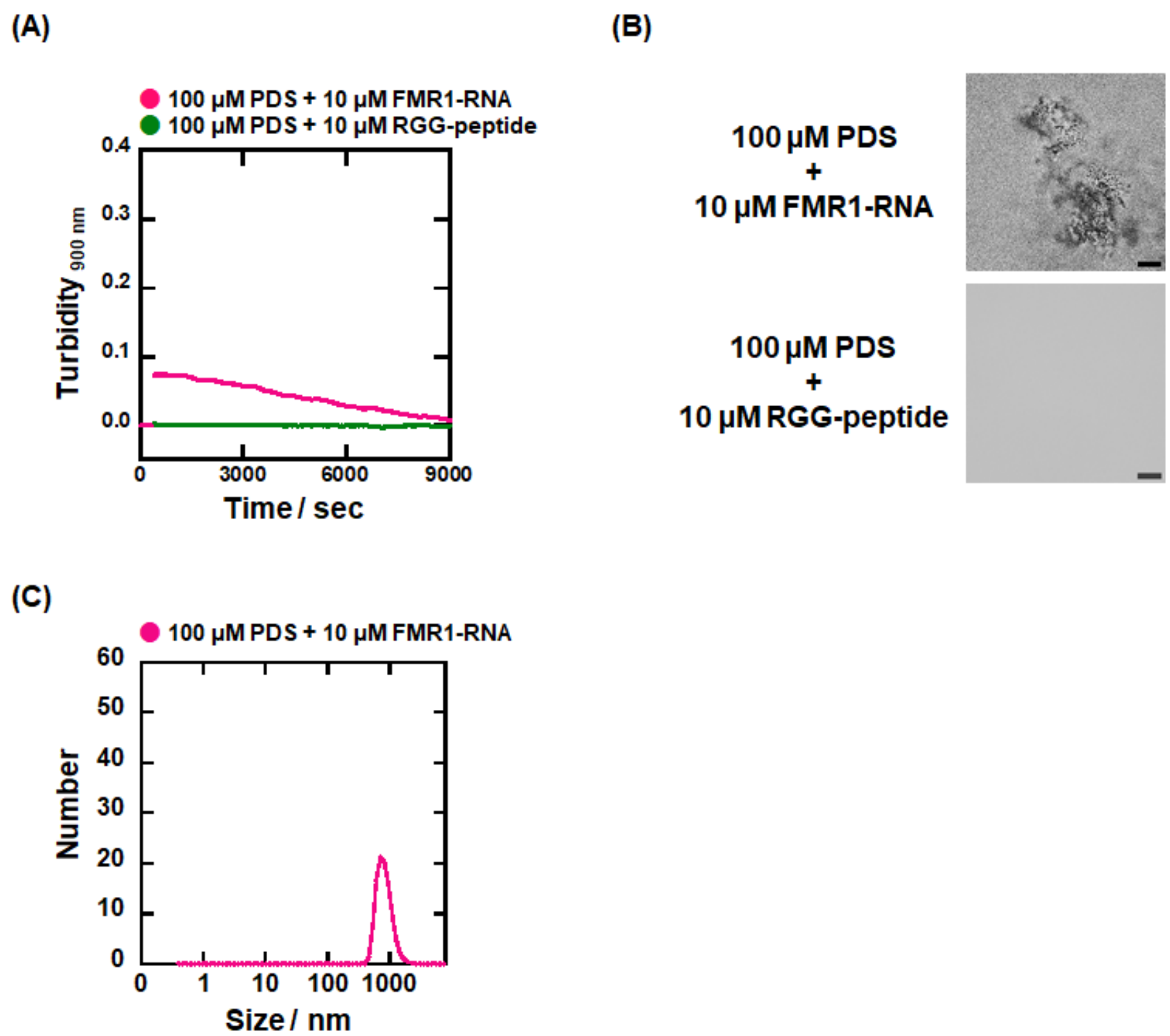

Figure S6. (A) Turbidity changes at 900 nm for the mixtures of 10 μM FMR1-RNA (red) or 10 μM RGG-peptide (green) with 100 μM PDS in the buffer containing 100 mM KCl and 50 mM MES-LiOH (pH 7.0) at 25 °C. (B) Bright field images for the mixtures of 10 μM FMR1-RNA (top) or 10 μM RGG-peptide (bottom) with 100 μM PDS after 3600 s incubation at room temperature. Scale bar = 10 μm. (C) DLS of the mixture of 10 μM FMR1-RNA and 10 μM RGG-peptide after 3600 s incubation at 25 °C in the buffer containing 100 mM KCl and 50 mM MES-LiOH (pH 7.0) at 25 °C.

(A)

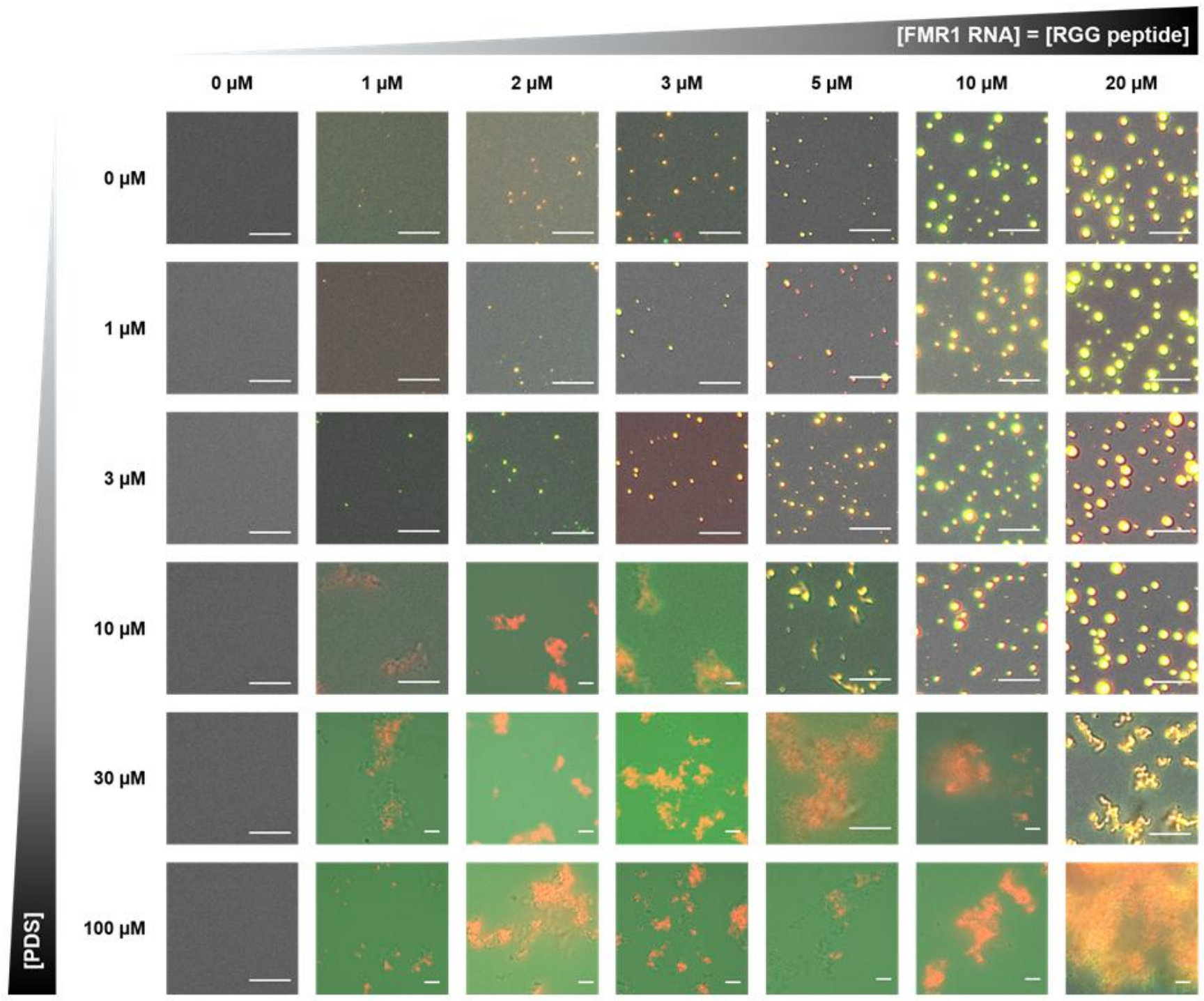

(B)

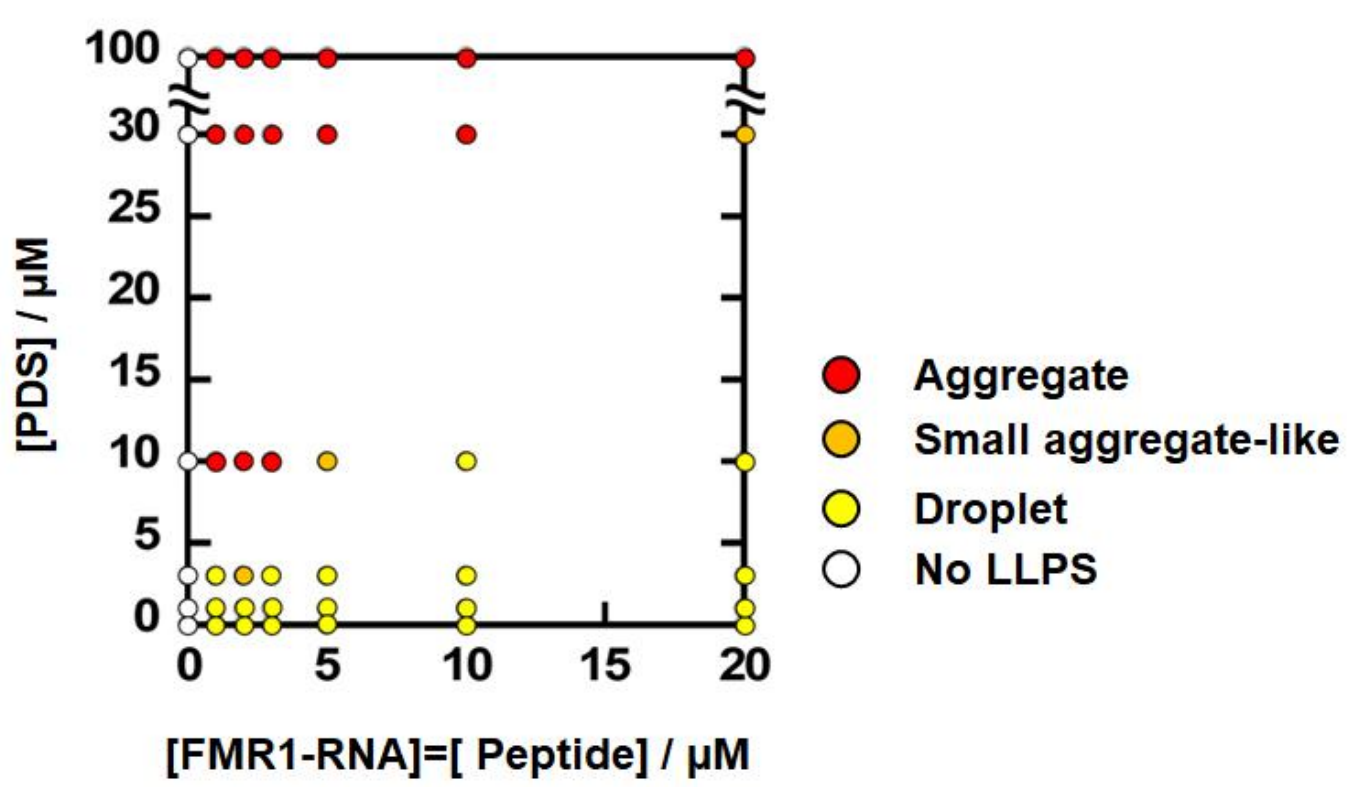

Figure S7. (A) Merged fluorescence images of C-FMR1-RNA and F-RGG-peptide in mixtures containing various concentrations of C-FMR1-RNA and F-RGG-peptide with various concentrations of PDS in the buffer containing 100 mM KCl and 50 mM MES-LiOH (pH 7.0) after incubation for 3600 s at room temperature. Scale bars = 10 µm. (B) Phase diagram of the mixture of equimolar of C-FMR1-RNA and F-RGG-peptide at various concentrations of PDS. Aggregates, small aggregate-like structures, droplets and no droplet formation are indicated in red, orange, yellow and white, respectively.

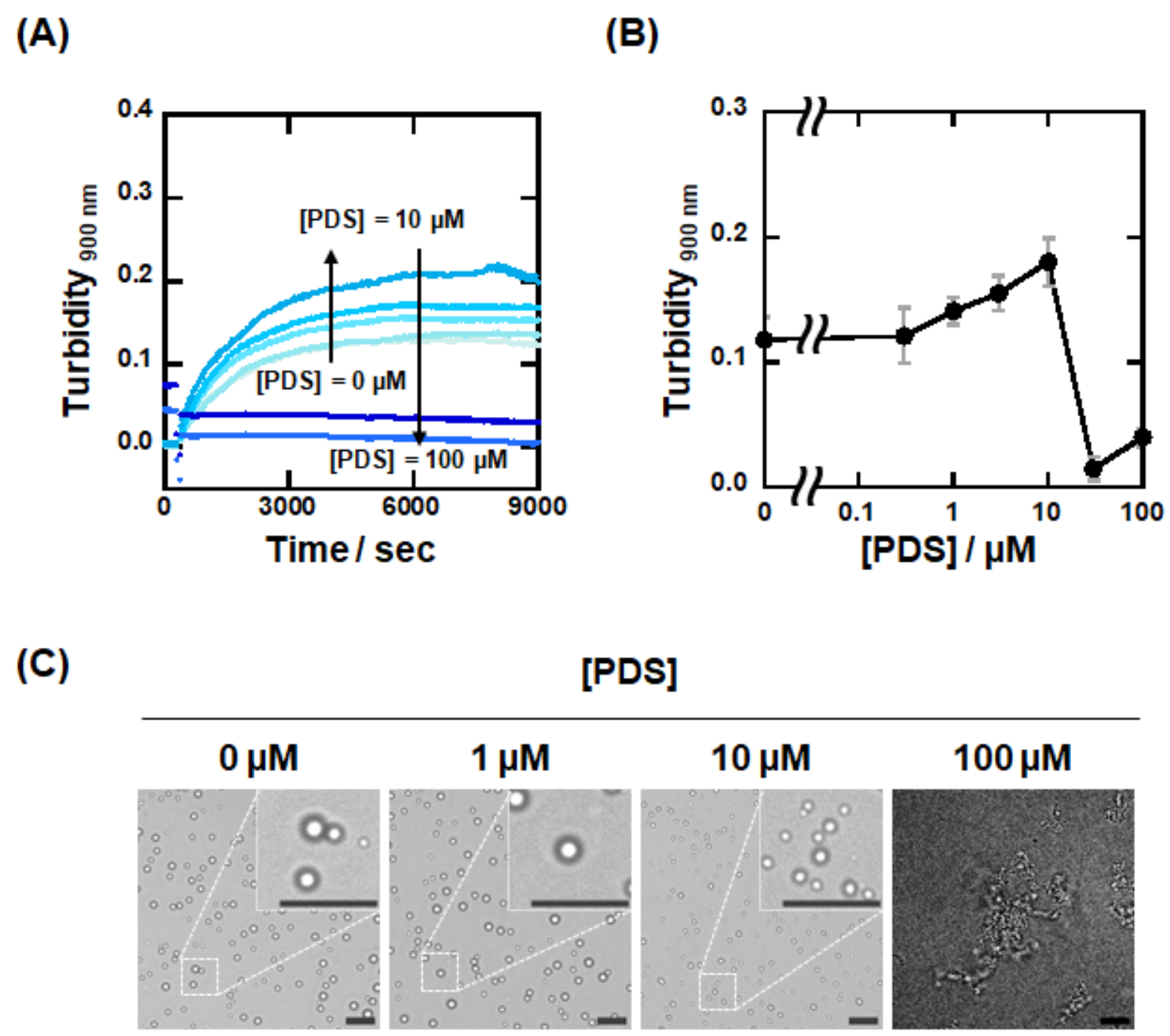

Figure S8. (A) Turbidity changes at 900 nm for the mixture of 10 µM C9orf72-RNA and 10 µM RGG-peptide with various concentrations (0, 0.3, 1, 3, 10, 30 and 100 µM) of PDS in the buffer containing 100 mM KCl and 50 mM MES-LiOH (pH 7.0) at 25 °C. (B) Plots of turbidity at 900 nm for mixture of 10 µM C9orf72-RNA and 10 µM RGG-peptide in the presence of various concentrations of PDS after incubation for 3600 s. (C) Bright field images of mixtures of 10 µM C9orf72-RNA or 10 µM RGG-peptide with various concentrations (0, 1, 10 and 100 µM) of PDS after incubation for 3600 s at room temperature. Scale bar = 10 µm.

PhenDC3

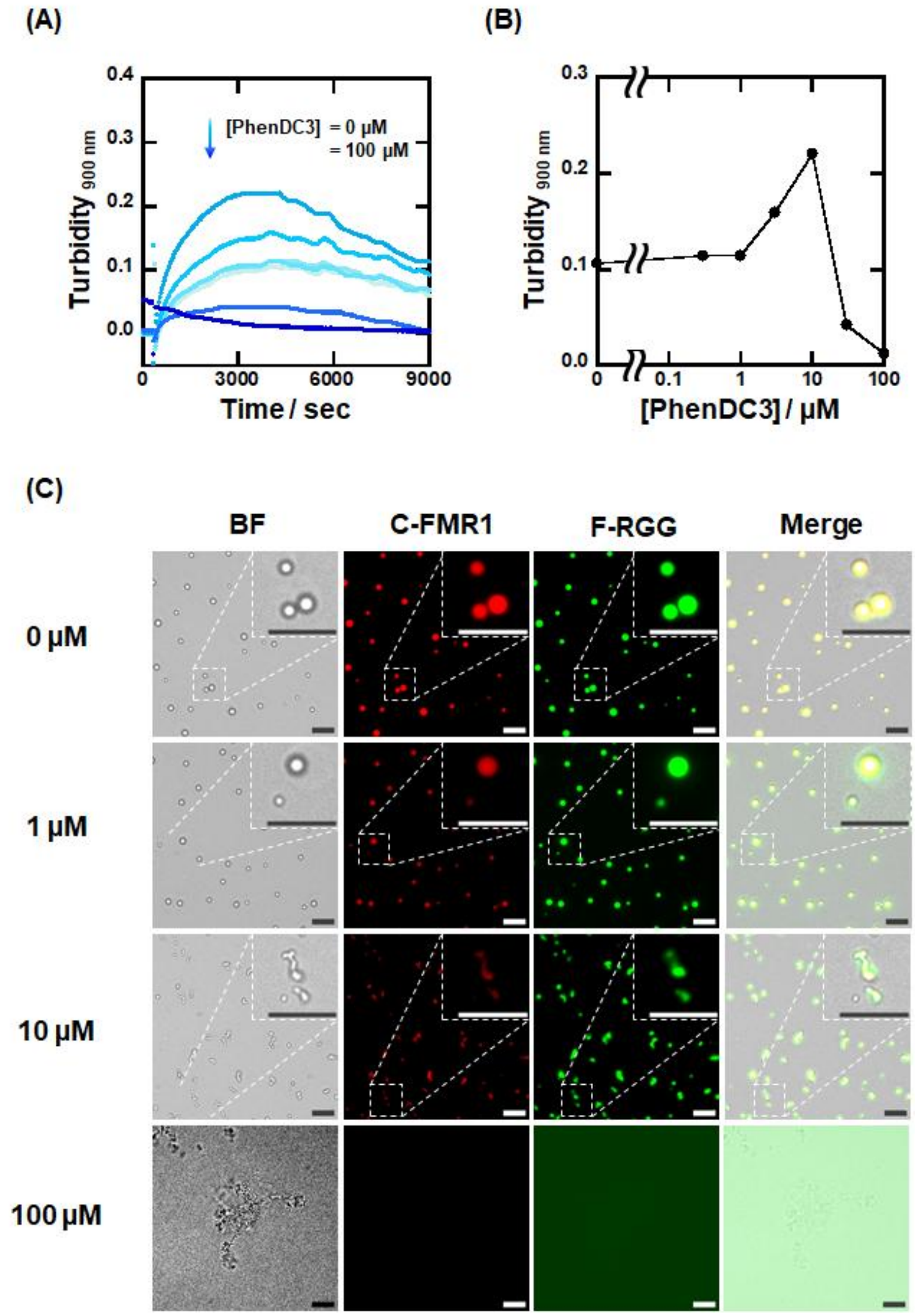

Figure S9. (A) Turbidity changes at 900 nm for mixtures of 10 µM FMR1-RNA and 10 µM RGG-peptide with various concentrations (0, 0.3, 1, 3, 10, 30 and 100 µM) of PhenDC3 at 25 °C. (B) Plots of turbidity at 900 nm for mixture of 10 µM FMR1-RNA and 10 µM RGG-peptide in the presence of various concentrations of PhenDC3 after incubation for 3600 s. (C) Fluorescence microscopy images showing mixtures of 10 µM C-FMR1-RNA and 10 µM F-RGG-peptide with various concentrations of PhenDC3 (0, 1, 10 and 100 µM) after incubation for 3600 s at room temperature. Scale bar = 10 µm. All experiments were carried out in a buffer containing 100 mM KCl and 50 mM MES-LiOH (pH 7.0).

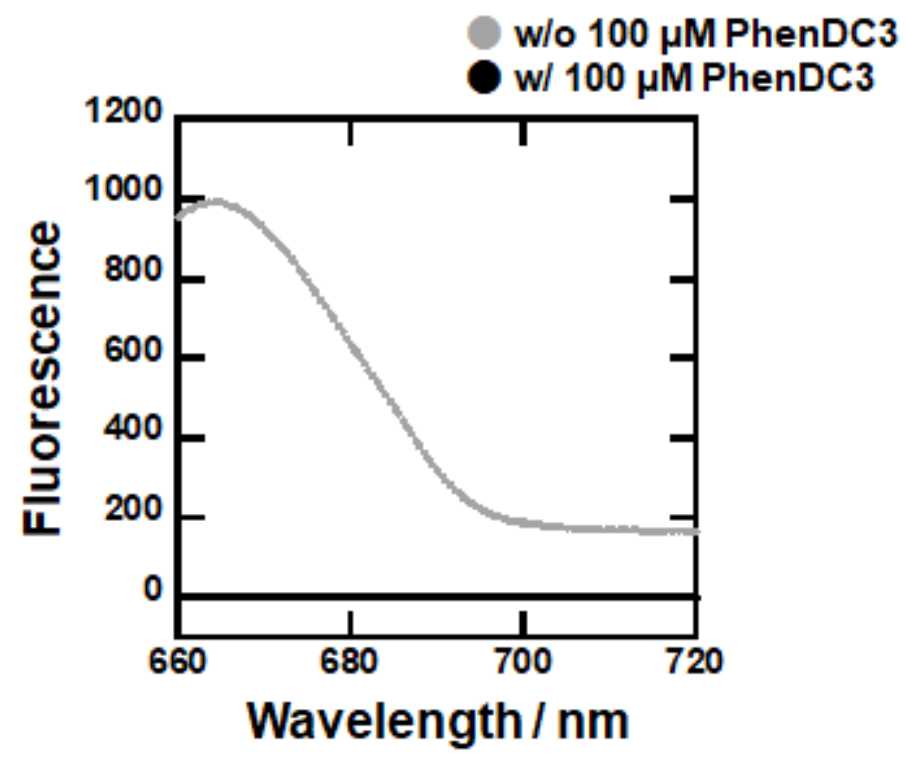

Figure S10. Fluorescence spectra of 1 µM C-FMR1-RNA in the absence (gray) or presence (black) of 100 µM PhenDC3 in the buffer containing 100 mM KCl and 50 mM MES-LiOH (pH 7.0) at 25 °C.

TMPyP4

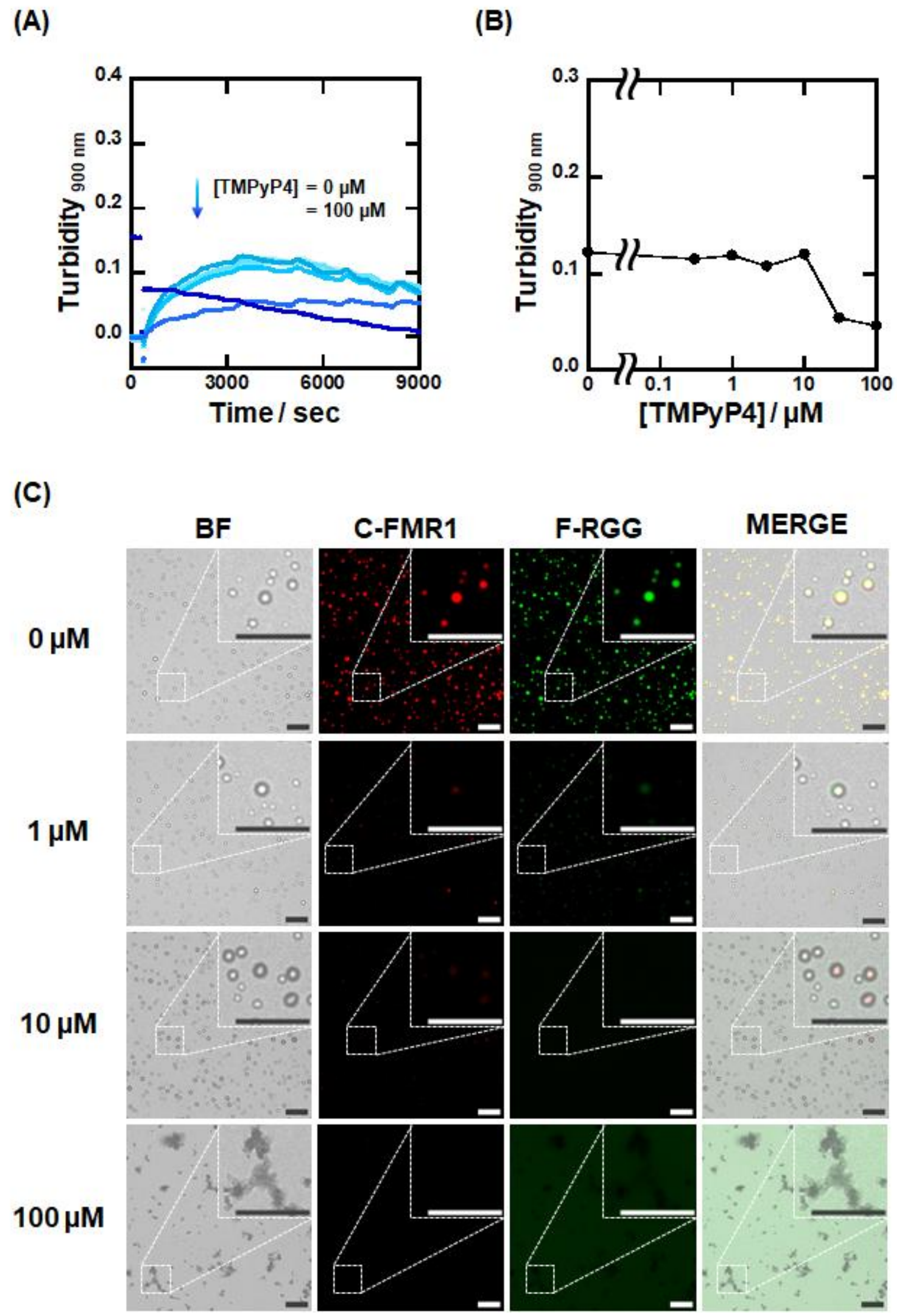

Figure S11. (A) Turbidity changes at 900 nm for mixtures of 10 μM FMR1-RNA and 10 μM RGG-peptide with various concentrations (0, 0.3, 1, 3, 10, 30 and 100 μM) of TMPyP4 at 25 °C. (B) Plots of turbidity at 900 nm for mixture of 10 μM FMR1-RNA and 10 μM RGG-peptide in the presence of various concentrations of TMPyP4 after incubation for 3600 s. (C) Fluorescence microscopy images showing mixtures of 10 μM C-FMR1-RNA and 10 μM F-RGG-peptide with various concentrations of TMPyP4 (0, 1, 10 and 100 μM) after incubation for 3600 s at room temperature. Scale bar = 10 μm. All experiments were carried out in a buffer containing 100 mM KCl and 50 mM MES-LiOH (pH 7.0).

ThT

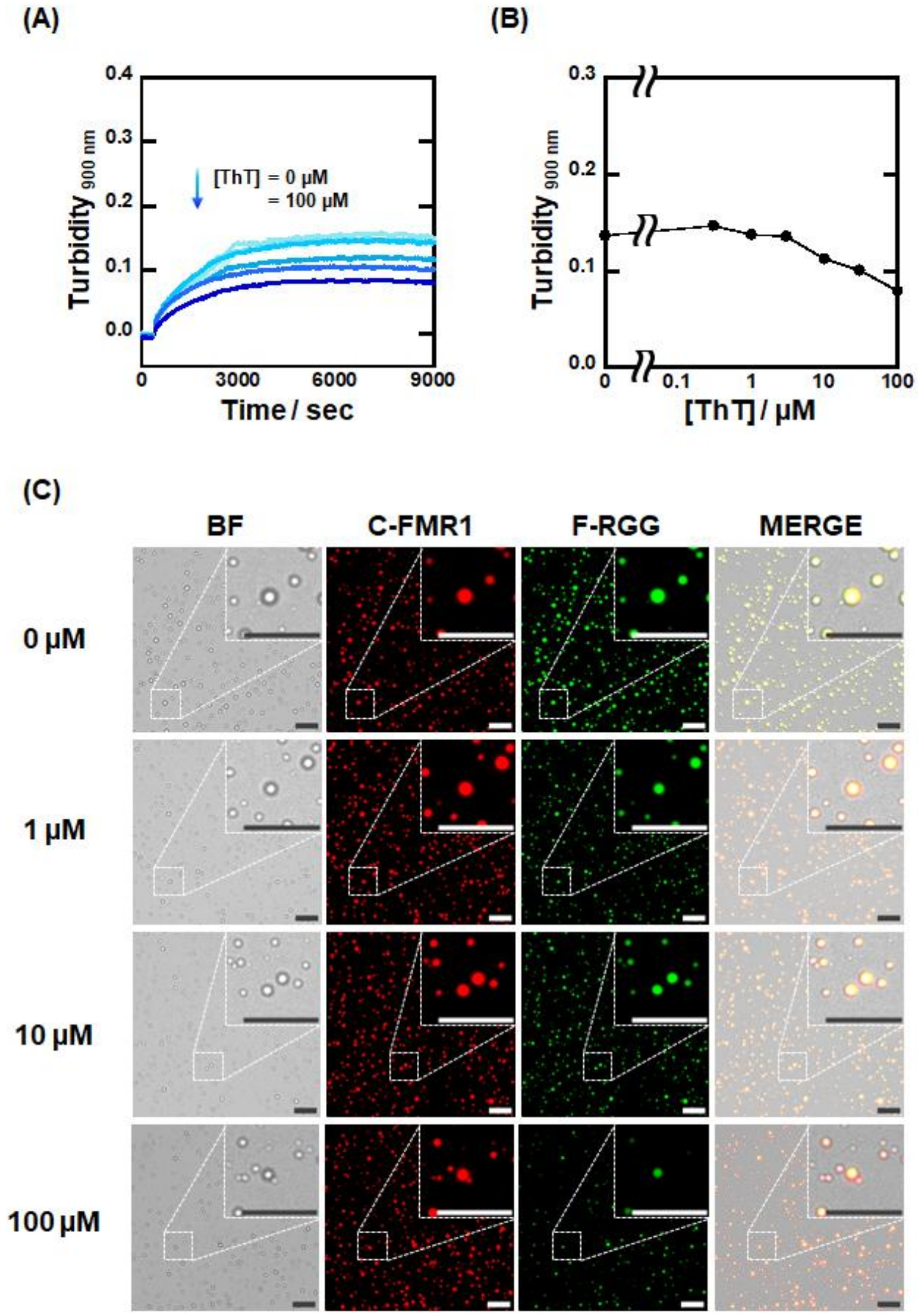

Figure S12. (A) Turbidity changes at 900 nm for mixtures of 10 μM FMR1-RNA and 10 μM RGG-peptide with various concentrations (0, 0.3, 1, 3, 10, 30 and 100 μM) of ThT at 25 °C. (B) Plots of turbidity at 900 nm for mixture of 10 μM FMR1-RNA and 10 μM RGG-peptide in the presence of various concentrations of ThT after incubation for 3600 s. (C) Fluorescence microscopy images showing mixtures of 10 μM C-FMR1-RNA and 10 μM F-RGG-peptide with various concentrations of ThT (0, 1, 10 and 100 μM) after incubation for 3600 s at room temperature. Scale bar = 10 μm. All experiments were carried out in a buffer containing 100 mM KCl and 50 mM MES-LiOH (pH 7.0).

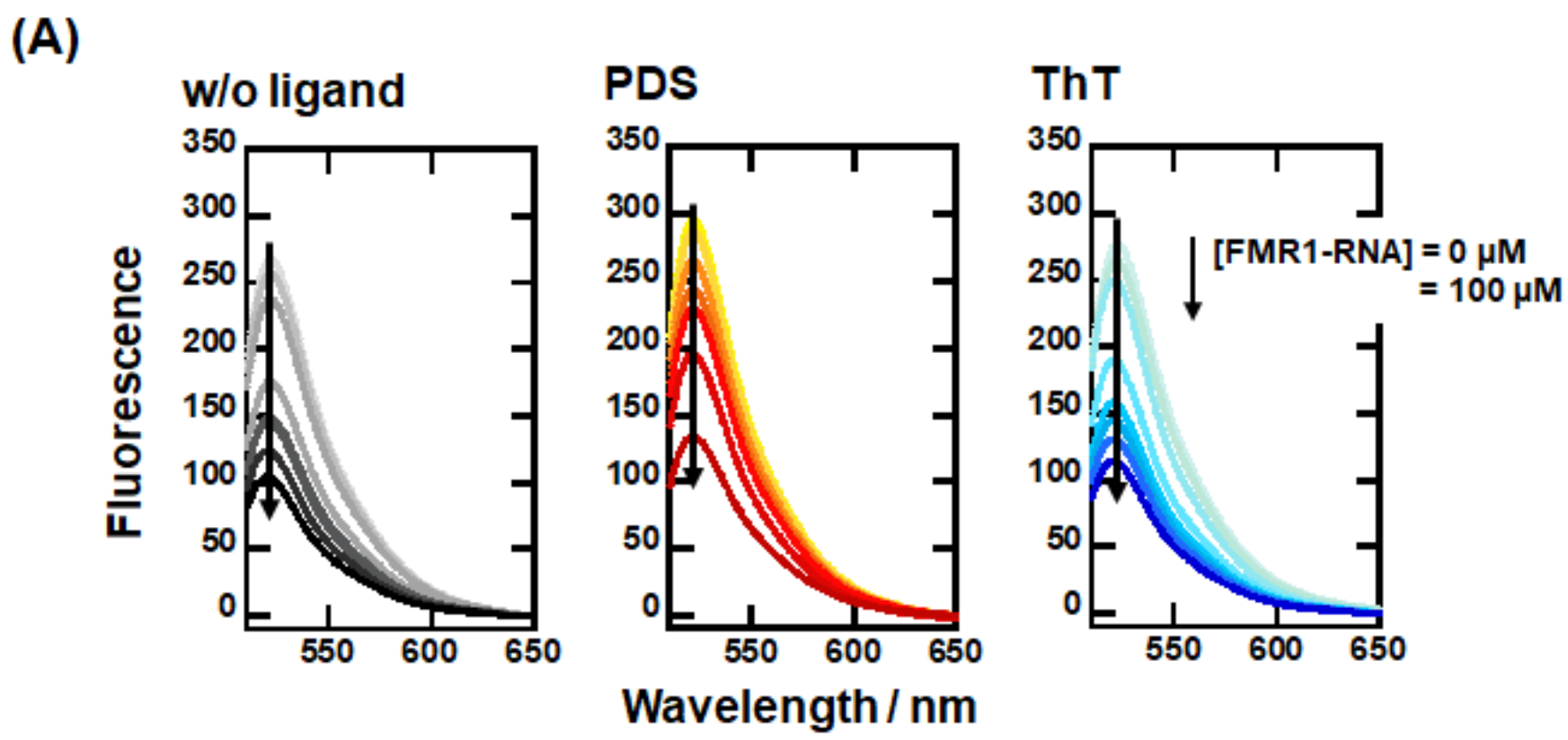

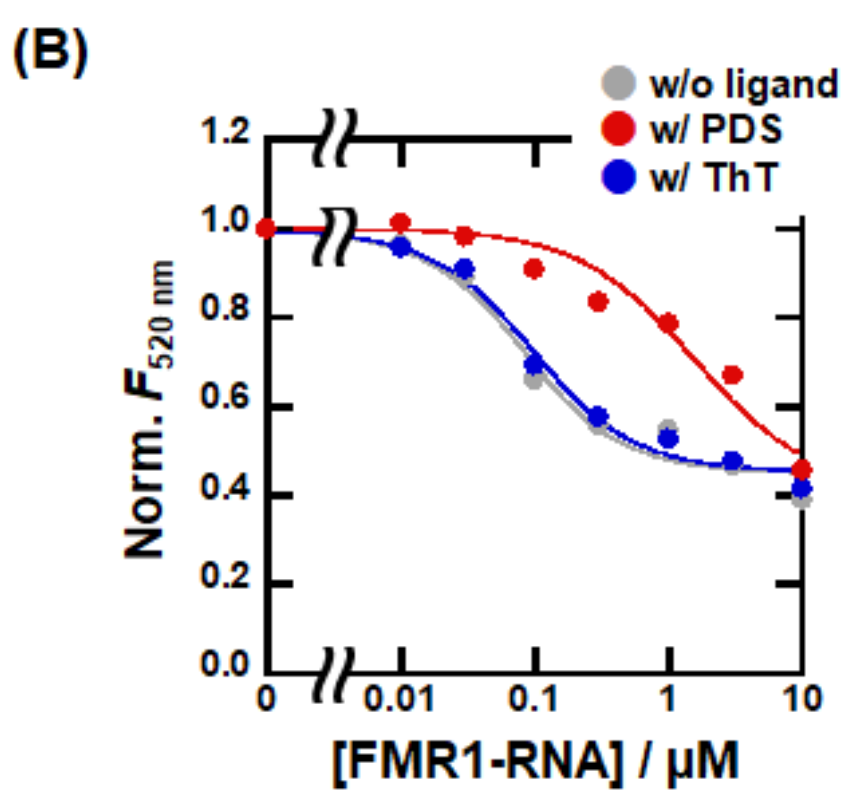

(C)

	K_d (µM)
w/o ligand	8.0×10^{-2}
w/ PDS	3.0
w/ ThT	9.4×10^{-2}

Figure S13. (A) Fluorescence spectra of 50 nM F-RGG-peptide with increasing concentrations of FMR1-RNA (0, 0.01, 0.03, 0.1, 0.3, 1, 3 and 10 µM) in the absence of G4 ligand (left) or in the presence of 100 µM PDS (center) or ThT (right) at 25 °C. The excitation wavelength was 495 nm. (B) Plots of normalized fluorescence at 520 nm of 50 nM F-RGG-peptide versus the concentration of FMR1-RNA at 25 °C. (C) The K_d values of F-RGG-peptide with FMR1-RNA in the absence of G4 ligand or in the presence of 100 µM PDS or ThT at 25 °C. All experiments were carried out in the buffer containing 100 mM KCl and 50 mM MES-LiOH (pH 7.0).

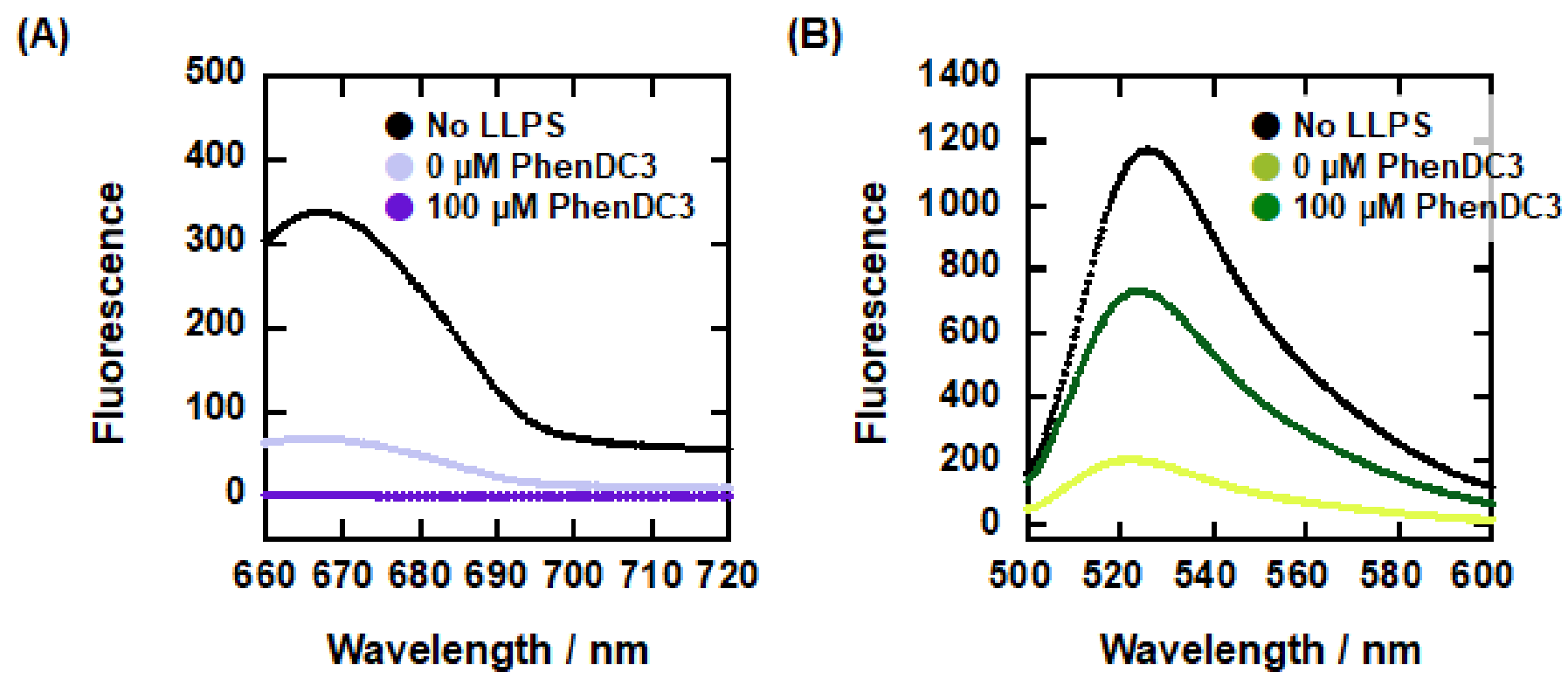

Figure S14. Fluorescence spectra of C-FMR1-RNA (A) and F-RGG-peptide (B). In panel A, the black, light purple, and dark purple traces represent the sample in solution, LLPS samples formed in the absence of PhenDC3, and LLPS samples formed in the presence of 100 µM PhenDC3, respectively. In panel B, the black, light green, and dark green traces represent the sample in solution, LLPS samples formed in the absence of PhenDC3, and LLPS samples formed in the presence of 100 µM PhenDC3, respectively. All measurements were performed in a buffer containing 100 mM KCl and 50 mM MES-LiOH (pH 7.0) at 25 °C.

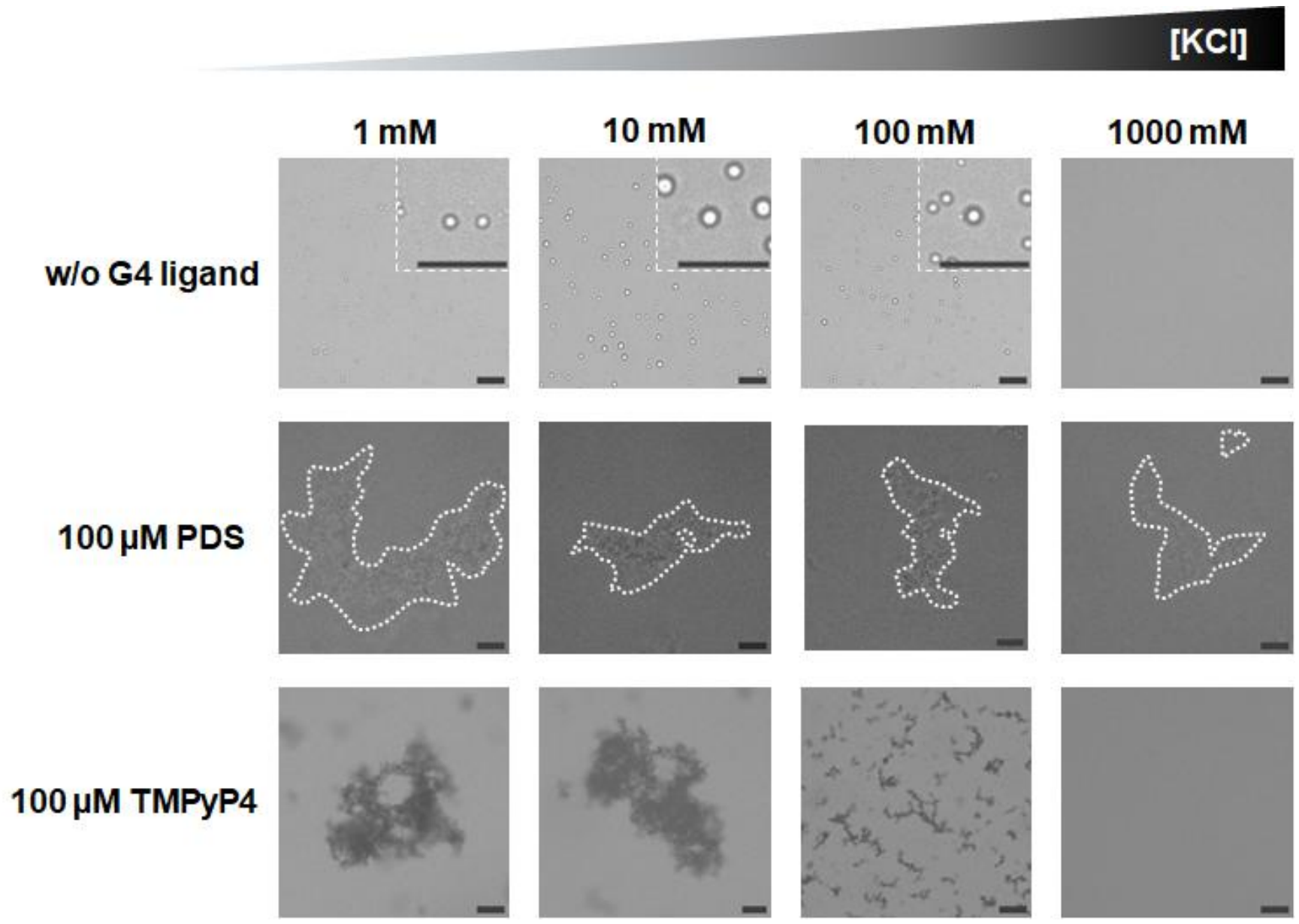

Figure S15. Bright field images of the mixture of 10 µM FMR1-RNA and 10 µM RGG-peptide without G4-ligand (top), with 100 µM PDS (middle) or TMPyP4 (bottom), at 1 mM, 10 mM, 100 mM or 1000 mM KCl (from left to right) in the buffer containing 50 mM MES-LiOH (pH 7.0) after incubation for 3600 s at room temperature. The aggregates observed in the presence of 100 µM PDS are outlined with white dashed lines for clarity.

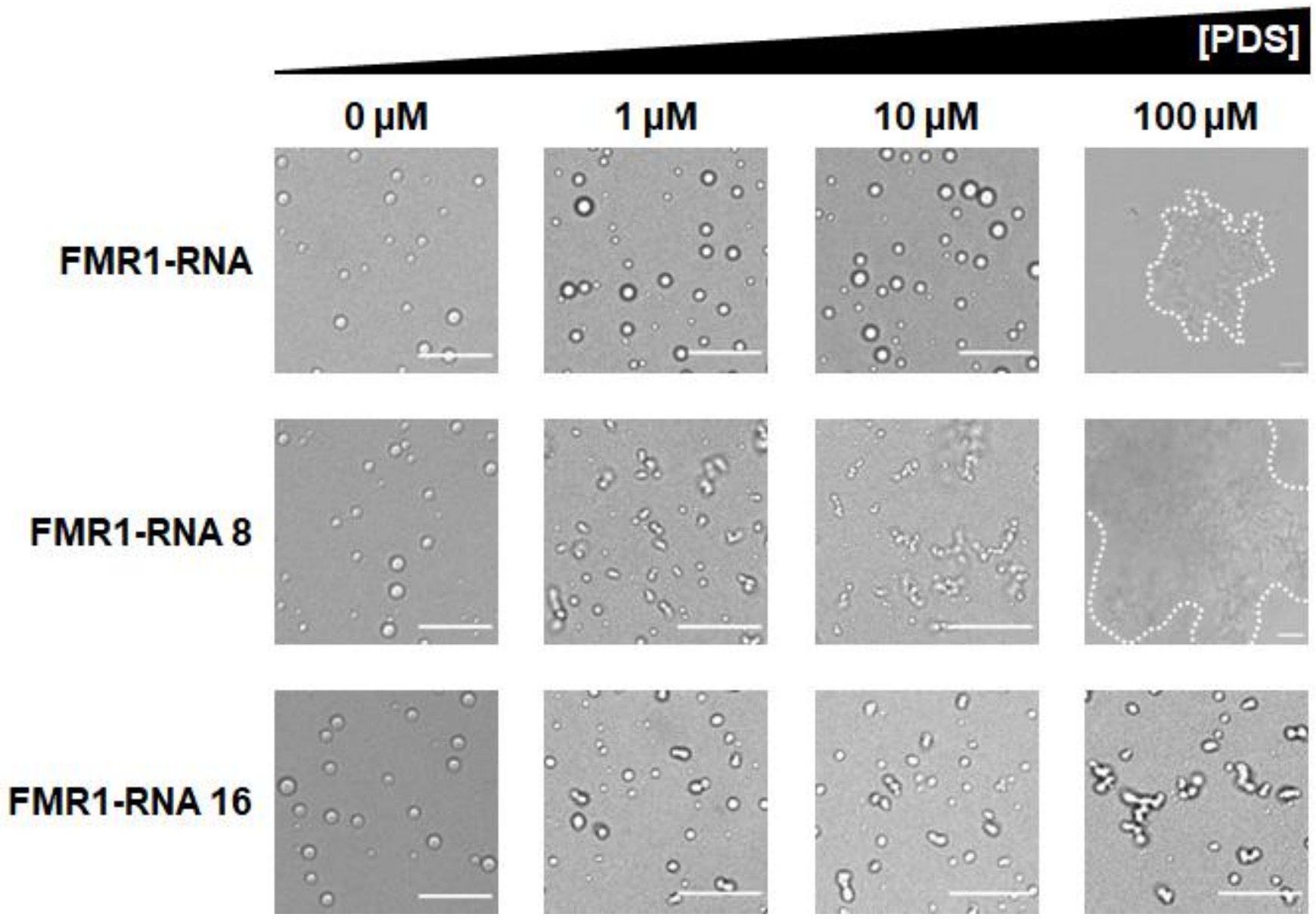

Figure S16. Bright field images of mixtures containing 10 μM FMR1-RNA, 5 μM FMR1-RNA 8, or 2.5 μM FMR1-RNA 16 and 10 μM RGG peptide in the presence of PDS (0, 1, 10 and 100 μM) in the buffer containing 100 mM KCl and 50 mM MES-LiOH (pH 7.0) after incubation for 3600 s at room temperature. The aggregates observed in the presence of 100 μM PDS are outlined with white dashed lines for clarity.
